# Point-of-care tests in the emergency medical services: a scoping review

**DOI:** 10.1186/s13049-025-01329-y

**Published:** 2025-02-03

**Authors:** T. H. M. Moore, S. Dawson, K. Kirby, R. Body, A. Thompson, Y. O. Adepoju, R. Perry, H. Nicholson, J. Dinnes, K. Mitchell, J. Savović, S. Voss, J. R. Benger

**Affiliations:** 1 The National Institute for Health and Care Research Applied Research Collaboration West (NIHR ARC West) at University Hospitals Bristol and Weston NHS Foundation Trust, Bristol, UK; 2 Population Health Sciences, Bristol Medical School, University of Bristol, Bristol, UK; 3 School of Health and Social Wellbeing, University of the West of England, Bristol, UK; 4 South Western Ambulance Service NHS Foundation Trust, Bristol, UK; 5 Emergency Department, Manchester University NHS Foundation Trust, Manchester, UK; 6 Division of Cardiovascular Sciences, The University of Manchester, Manchester, UK; 7 Division of Population Health, Health Services Research & Primary Care, School of Health Sciences, University of Manchester, Manchester, UK; 8 Gloucestershire NHS Foundation Trust, Gloucester, UK; 9 Department of Applied Health Sciences, School of Health Sciences, University of Birmingham, Birmingham, UK; 10 The National Institute for Health and Care Research Applied Research Birmingham Biomedical Research Centre, University Hospitals Birmingham NHS Foundation Trust, University of Birmingham, Birmingham, UK

**Keywords:** Point-of-care tests, Diagnosis, Ambulance, Emergency medical services, Trauma, Sepsis, Acute coronary syndromes, Troponin, Lactate, Scoping review

## Abstract

**Background:**

This scoping review aimed to summarize existing research on point-of-care tests (POCTs) within emergency medical services (EMS). There is a lack of comprehensive reviews covering the breadth and scope of application of POCTs in EMS despite growing interest and potential benefits in this setting. A review of the research will inform how we target future research efforts to support effective implementation and avoid duplication.

**Methods:**

We searched three databases to April 2023 using comprehensive terms for POCTs. One author screened titles and abstracts, full-text papers and extracted data with a second author checking the data. A scoping review framework was used to categorise studies according to demographics, study design, medical conditions, biomarkers and test devices.

**Results:**

We found 141 papers that included 158 reports of 9 study designs, 155 reports of 40 combinations of biomarker and condition and 161 reports of 41 test-devices. The majority of research was done in the UK (19%), US (17%), and the Netherlands (16%), mostly in land-based EMS (82%). Most frequently assessed were troponin for acute coronary syndromes (26%), lactate for sepsis (14%) or lactate for trauma/critical illness (13%). The majority of research designs investigated the accuracy of the tests (43%). Few studies were of a design to inform guidelines to change patient pathways and the associated outcomes, including, randomised controlled trials (RCTs) (4%), non-randomised studies able to assess causality (6%), economic analyses (1%) or qualitative work on acceptability (3%). In those few cases where RCTs were done there were long delays between initial test-accuracy research and publication of the first RCT, for example 11 years delay for troponin for acute coronary syndromes.

**Conclusions:**

We identified a thriving base of research on POCT in the EMS, however most studies established the diagnostic accuracy of the tests with few RCTs, economic analyses or qualitative research on acceptability. The time-lag from diagnostic accuracy to developing an RCT is considerable. Investment in funding and infrastructure is needed to support the research pathway for potential POCTs beyond diagnostic accuracy to designs able to assess clinical effectiveness, acceptability and economic effectiveness.

**Supplementary Information:**

The online version contains supplementary material available at 10.1186/s13049-025-01329-y.

## Introduction

A point-of-care test (POCT) refers to medical diagnostic testing performed near the patient, typically at the point of care rather than in a centralized laboratory setting. A POCT offers rapid results, enabling timely clinical decision-making, particularly in settings where immediate action is crucial, such as emergency medical services (EMS) [1]. Over the past few decades, advancements in technology such as microfluidics, miniaturization, nanoparticle techniques, multiplexing, wireless connectivity, and novel biomarkers have facilitated the development of portable and user-friendly POCT devices, expanding their applications across various healthcare settings such as primary care, the emergency department and EMS [2, 3]. POCTs delivered by EMS clinicians have potential to facilitate effective decision-making at scene that may support alternatives to conveyance to hospital [4], allow the initiation of correct treatments at an earlier stage, improve clinical outcomes [4, 5] and ensure patients are conveyed or directed to the facility or service that is best suited to their needs. In these ways patient pathways could be reconfigured to increase system capacity, reduce bottle necks, improve outcomes and enhance patient and carer experience.

Despite the potential benefits of POCTs in EMS, we were unable to find any systematic reviews summarising the current breadth and scope of research where POCTs are in use, or have potential use in the EMS setting. We did identify reviews on POCTs for acute coronary syndromes (ACS) mostly using troponin [6–11]; testing of trauma patients [12–18], and lactate testing for sepsis [19–23]. One systematic review of panel tests in the EMS and emergency department identified only one EMS study [24]. Other scoping reviews on POCTs exist but have focussed on primary care [25]. To support the successful commissioning and implementation of POCTs in EMS, policy makers require evidence from well-conducted randomised controlled trials (RCTs) or non-randomised studies of interventions (NRSI) together with economic evaluations and information on acceptability from qualitative research. Researchers with an interest in optimising their experimental research on POCTs would, ideally, focus their efforts to avoid unnecessary duplication. The aim of this scoping review is to summarize published research into POCTs in EMS, according to the conditions and biomarkers being assessed, the research designs and test devices being used.

## Methods

Scoping reviews identify the types of evidence in a given field, and explore and assess knowledge gaps [26]. We followed published methods for conducting and reporting scoping reviews and reported according to the PRISMA extension for scoping reviews [27–30]. The review protocol is available online [31]. This review informed a research project that used a multiple criteria decision analysis (MCDA) to identify candidate POCTs and use cases for a platform trial of POCTs in UK EMS [32]. In order to meet deadlines related to the grant applications we adopted a rapid review approach, expediting screening processes and increasing specificity of the search.

### Eligibility criteria

We included studies evaluating the use of a POCT in the EMS. Eligible sample types for the POCT included any bodily fluids (saliva, blood, serum, exhaled breath condensate, sweat). Any study design was eligible including, but not limited to, systematic reviews, RCTs, controlled trials, comparative cohort studies, single arm cohort studies (labelled as observational studies), qualitative studies and any design looking at diagnostic test accuracy. We excluded case studies, prevalence studies, or protocols for managing disease, studies evaluating imaging tests such as ultrasound, and specialist services such as mobile stroke units because of the reliance on specialist personnel and scanning equipment in conjunction with the POCT.

### Search strategy

We searched electronic databases (MEDLINE and EMBASE via Ovid and CINAHL on Ebscohost) to July 2023. The searches were developed by an information specialist (SD) in liaison with the rest of the team. The initial search was broad, combining terms for < point-of-care tests > AND < emergency medical services>. This was supplemented with targeted searches for specific biomarkers and conditions following meetings with stakeholders as part of the MCDA who identified six biomarkers considered to have potential for use in the EMS (troponin, NT-ProBNP, lactate, beta-hCG, head trauma biomarkers, ketones) and POCT for any respiratory tract infection [33]. Stakeholders contributing to the discussion are listed and acknowledged in the MCDA paper, and include the authors of this paper, they included public contributors with experience of using emergency medical services, consultants in medical biochemistry and pharmacology, infectious diseases, intensive care, emergency medicine, research paramedics and leads of clinical ambulance services [33]. We did not apply a filter for types of study design. We searched reference lists of key systematic reviews. We restricted to English language and research published after 2000, since the technologies supporting POCT in EMS are developing and improving rapidly. A table summarising the searches is presented in the supplemental material and on Open Science Framework [34].

### Study selection and data collection and management

Titles and abstracts of records were screened for retrieval by one author (TM, SD, YA, RP, HN, JD) with all excluded records checked for exclusion by a second author (TM, SD, YA, RP, JD, HN). Full-text versions were screened in the same way. Data extraction was done by one author (TM) and checked by a second (KM, RP). Search data were managed using Rayyan and Endnote software (Ouzzani, 2016, The EndNote Team, 2013). Screening and data extraction were managed using the Microsoft software Access and Excel.

Data extracted included: type of test(s); test device name and manufacturer; study design; condition the test was being used for; biomarker being tested. Following convention in scoping reviews, methodological quality or risk of bias of included studies was not assessed as the purpose of this scoping review was to describe the breadth of evidence on POCT in EMS, rather than to synthesise evidence on effectiveness or diagnostic accuracy (Peters 2015).

### Data interpretation and presentation

Information extracted was organised into a framework of factors to understand the scope of the literature as described in Arksey and O’Malley and Peters et al. [27–29]. Categories included:

1. Demographics of the studies in terms of country of origin, type of publication, date, type of study, condition, biomarker and test.
2. Study design. Studies were categorised into one of eight potential designs: systematic review or systematic scoping review; randomised controlled trial; non-randomised study of intervention (NRSI) (comparative cohort study or controlled before and after studies); observation studies (cohort studies with a single group); diagnostic test accuracy studies (DTA) (studies comparing one or more diagnostic tests to a reference standard to determine diagnostic test accuracy such as positive predictive value, area under a ROC curve or sensitivity or specificity); qualitative studies; surveys; developmental study (e.g. a laboratory based study with the aim to see if a POCT could be developed for use in EMS, or setting based, e.g. mimicking shaking in helicopters or temperature variation).
3. Medical conditions, biomarkers and test devices. Initially we extracted the names of conditions, biomarkers and test devices as described in the papers. We then collapsed them into broader categories. For example, medical conditions such as septic shock, systemic inflammatory response syndrome and sepsis were all collapsed into one code “sepsis”. Chest pain (related to coronary artery disease), myocardial infarction, non-ST elevation acute myocardial infarction, non-ST elevation acute coronary syndrome were collapsed together as “acute coronary syndromes” (ACS). We had “condition” and “biomarker” as separate variables and a third variable for “combination of biomarker for condition”. Research reports on the use of POCT in general were labelled as “undifferentiated”. Research reports that encompassed many conditions or biomarkers were labelled as “multiple biomarkers” or “multiple conditions”. The categorisation of tests, biomarkers and conditions is provided in the supplemental material.

**Fig. 1:**
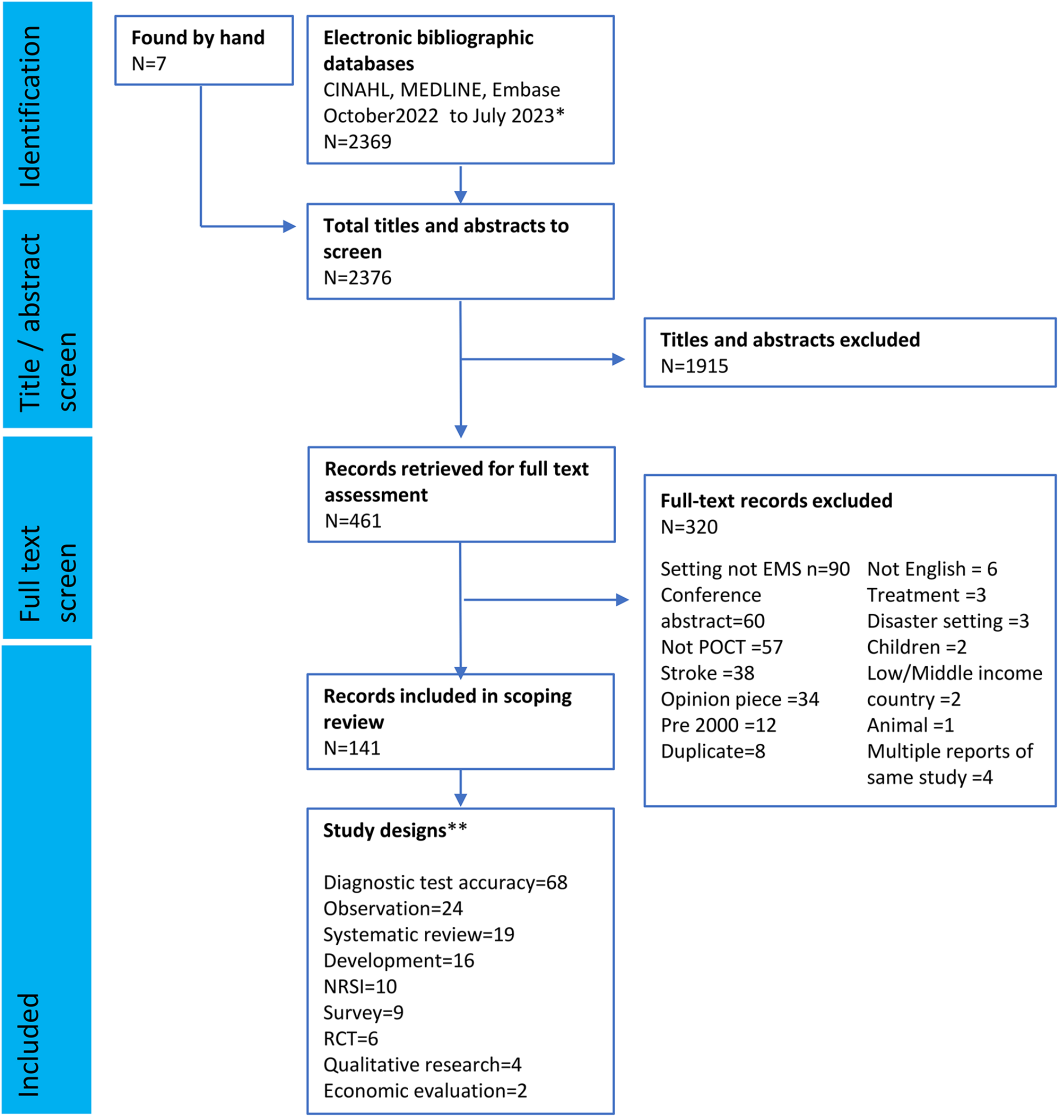
PRISMA flow diagram showing selection of studies into the review. Footnotes: *Details of search strategy are in supplementary material. **n = 158 different study designs reported in 141 papers, some studies included more than one study design. Abbreviations: RCT, Randomised controlled trial, NRSI, Non-randomised study of interventions

## Results

We retrieved a total of 2376 records from which 461 full-text papers were screened for inclusion. We excluded 320 records that did not meet the inclusion criteria. A flow chart of screening, including reasons for exclusion, is presented in Fig. 1. We included 141 papers that reported a total of 158 reports of nine study designs and 155 reports of 40 combinations of “biomarker for condition”. A full list of included records plus bibliographic information is in the supplementary data.

### Characteristics of included research

**Fig. 2:**
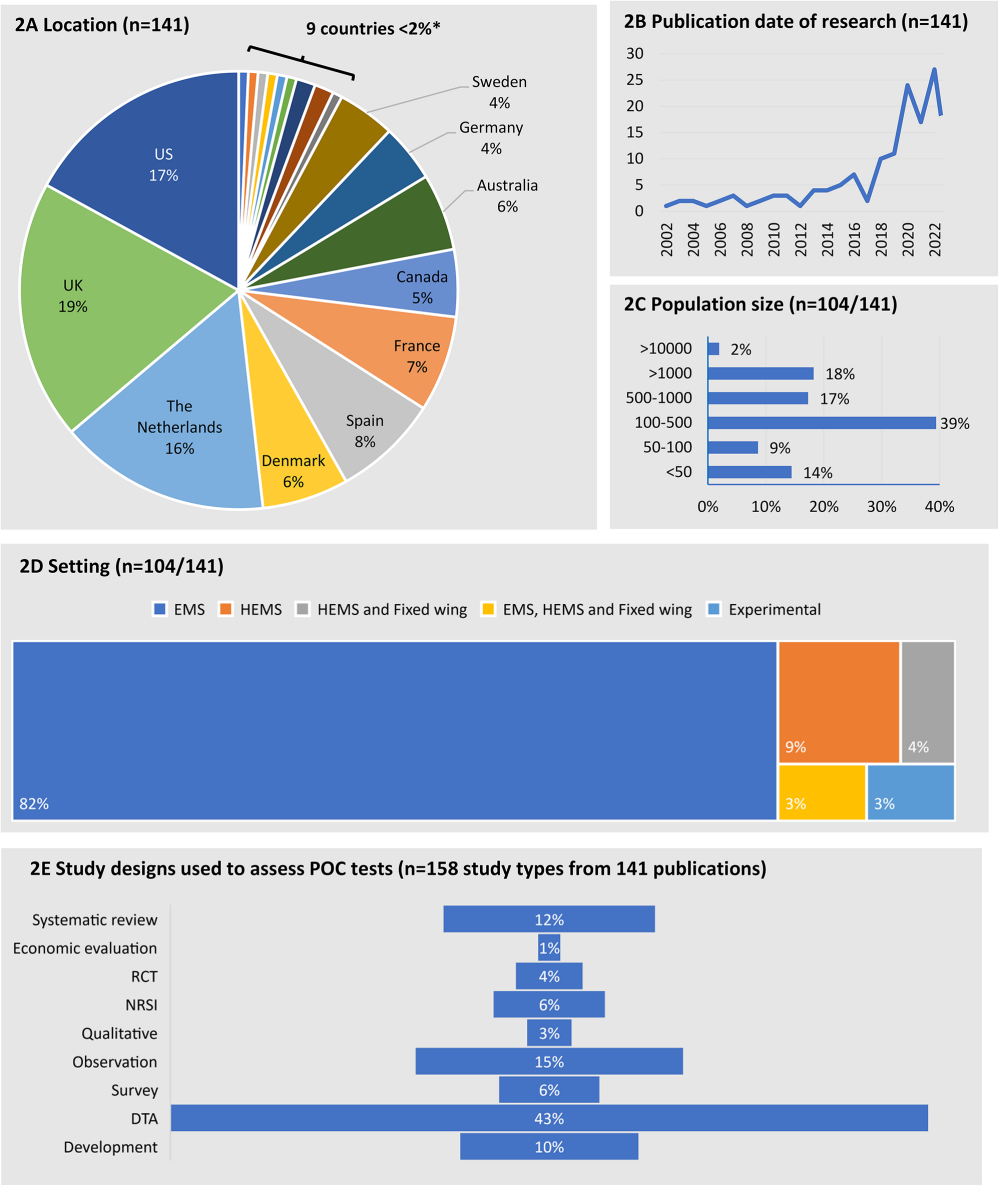
Characteristics of included research reports. Footnotes. 2 **A**) Countries with 2% or fewer studies were Finland and Italy (2 studies): Austria, Hungary, Japan, Norway, Slovenia, Taiwan, Switzerland (1 study). **2**B) Publication year of records. **2**D) EMS = Emergency medical services ground based; HEMS = Helicopter emergency medical services; Experimental = studies carried out in a laboratory but with the express purpose for using tests in the EMS. **2**E) Percentage of study types (*n* = 141 studies with *n* = 158 types of research reported). RCT Randomised controlled trial, NRSI non-randomised study of intervention, DTA Diagnostic test accuracy

More than half (73,52%) of the 141 papers were from three countries; the UK (27,19%), the US (24,17%) and The Netherlands (22,16%). Denmark, Spain, France and Australia each contributed between 5 and 8% (Fig. 2A). One study included more than one country in its scope, (Tran 2006) and this was a survey of POCT across the world. Lack of multi-country research might be a reflection on variation in organisation and focus of EMS. Most (104,76%) of the research was published since 2017 (Fig. 2B). More than a third of reports included 100 to 500 participants/data points (41,37% (Fig. 2C). Sample sizes in primary studies ranged from fewer than 50 people/data points (15,14%) to more than 10,000 (2,2%). The two studies with more than 15,000 data points both investigated acute coronary syndromes and use of POCTs across national EMS services in Australia and Denmark [35, 36]. Studies with very few participants/data points were typically qualitative research or laboratory development research. Studies were mostly set in land-based EMS (116,82%) with 21 (16%) reports in the helicopter emergency medical services (HEMS), or fixed wing or a mix of air and ground (Fig. 2D).

### Research design

Most papers described one type of research study, but 11% (17/141) reported two studies, for example a DTA study might be reported with a survey (Fig. 2E). In total, 9 different research designs were reported 158 times in 141 research papers. The most frequently reported studies evaluated diagnostic test accuracy (68,43%). Six (6,4%) of the published reports described three RCTs evaluating troponin for ACS (ARTICA, PROACT 3 and PROACT4) [37–41]. Ten reports (6%) were non-randomised studies of interventions (NRSI). Nine of these evaluated troponin for ACS [42–50] and one evaluated lactate for trauma patients transported by HEMS [51]. We found two (1%) economic evaluations of troponin [35, 52] (Fig. 2E). Twenty-four (15%) observational studies (cohort studies with a single group of patients exposed to the POCT without a control group) (24,15%) and 16 (10%) early phase development studies were also identified. Developmental evaluations considered issues such as the operating temperature of the test devices, or shaking of machines, for example to mimic transport in a helicopter, aeroplane or land vehicle.

Systematic and scoping reviews accounted for 12% (*n* = 19) of study designs reported. A third of reviews (6/19) included settings beyond EMS alone including other emergency settings, such as intensive care units, emergency departments, primary care and walk-in centres [8–10, 17, 21, 24]. Lactate was the most reviewed biomarker included in nine systematic reviews covering its use for sepsis (*n* = 4) [19, 20, 22, 23]; trauma (*n* = 4) [12, 15, 17, 18], or critical illness (*n* = 1) [21] (Fig. 2E). Troponin for ACS was included in five reviews [6, 7, 9–11], and heart-type fatty acid binding protein (H-FABP) for ACS in one [8].

### Time frame for evaluation of POCT in the EMS

**Fig. 3:**
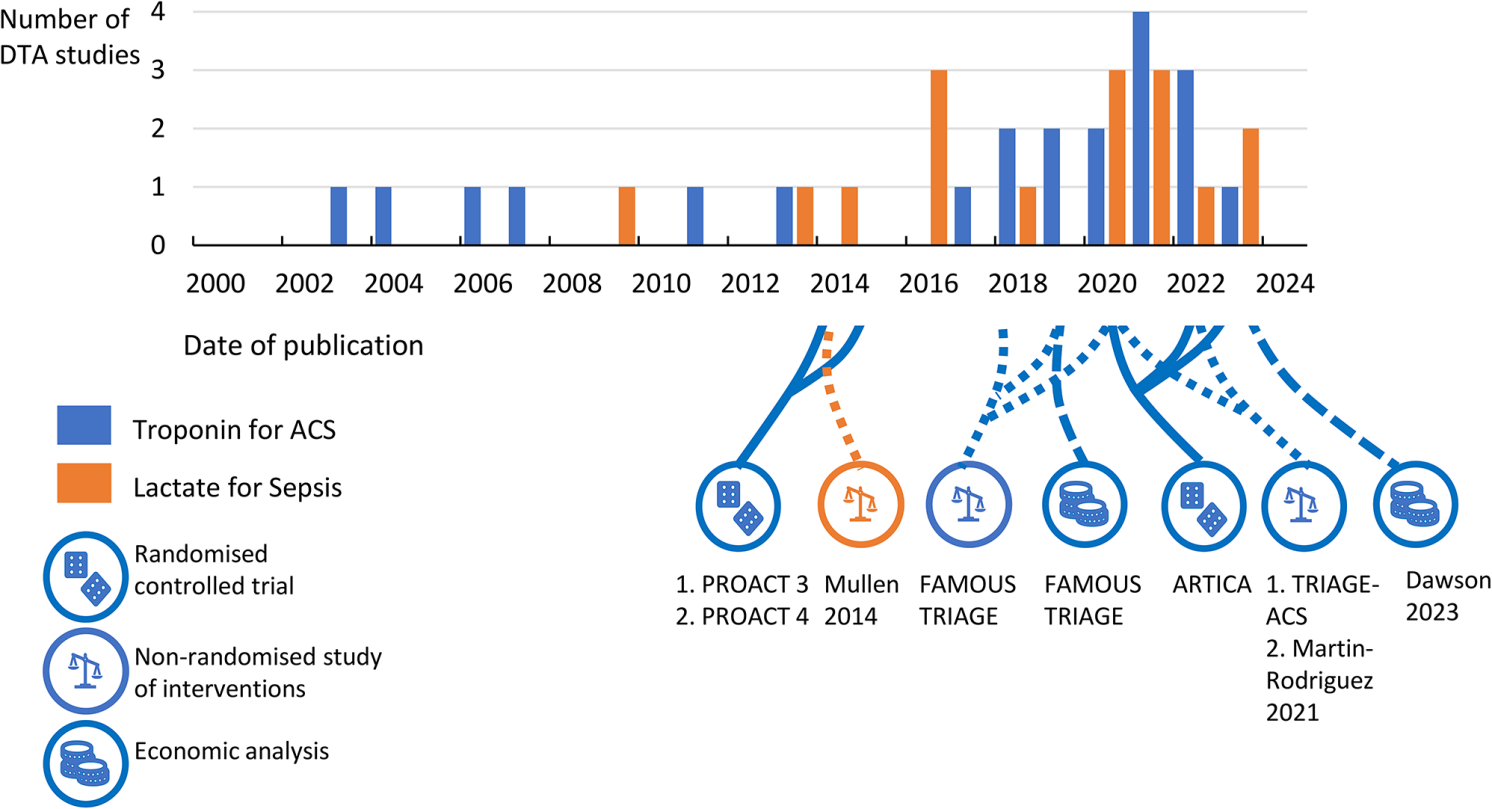
Timeline showing the number of diagnostic test studies (DTA) published per year and the relative publication dates of randomised controlled trials (RCT), non-randomised studies of interventions (NRSI) and economic analyses for two biomarker and condition combinations (a) Troponin for ACS and (b) Lactate for sepsis. Footnotes: ACS = Acute coronary syndromes. Number of diagnostic tests accuracy studies published per year are shown on a bar graph for the two biomarker/ condition combinations of (1) lactate for trauma and (2) troponin for ACS. Randomised controlled trials (RCTs) Economic analyses (EE) and Non-randomised studies of interventions (NRSI) are illustrated as icons below the bar graph showing their dates of publication. PROACT-3 [40]; PROACT 4 [41]; Mullen 2014 [51]; FAMOUSE TRIAGE [43, 46–50]; FAMOUS TRIAGE Economic evaluation [52]; ARTICA [37–39]; TRIAGE ACS [42]; Martin Rodriguz 2021 [44]; Dawson 2023 [35]

Taking lactate and troponin as examples with the most mature research landscapes, the evidence base demonstrates a dearth of RCTs, and where RCTs have been conducted, a considerable time lag of up to 11 years from initial research on test accuracy in EMS (Fig. 3). The first full-text research we identified on troponin in EMS, a diagnostic test accuracy study, was published over 20 years ago in 2003 [53]. The first RCT was published in 2014 [40] and the first comparative cohort analyses were published in 2018 [47]. A shorter duration of research development is apparent for lactate with the first research on diagnostic test accuracy in 2008 [54], and the first comparative effectiveness study in 2014 [51]. However at the date of the search for this review there were no RCTs.

### Biomarkers and conditions of interest

**Fig. 4:**
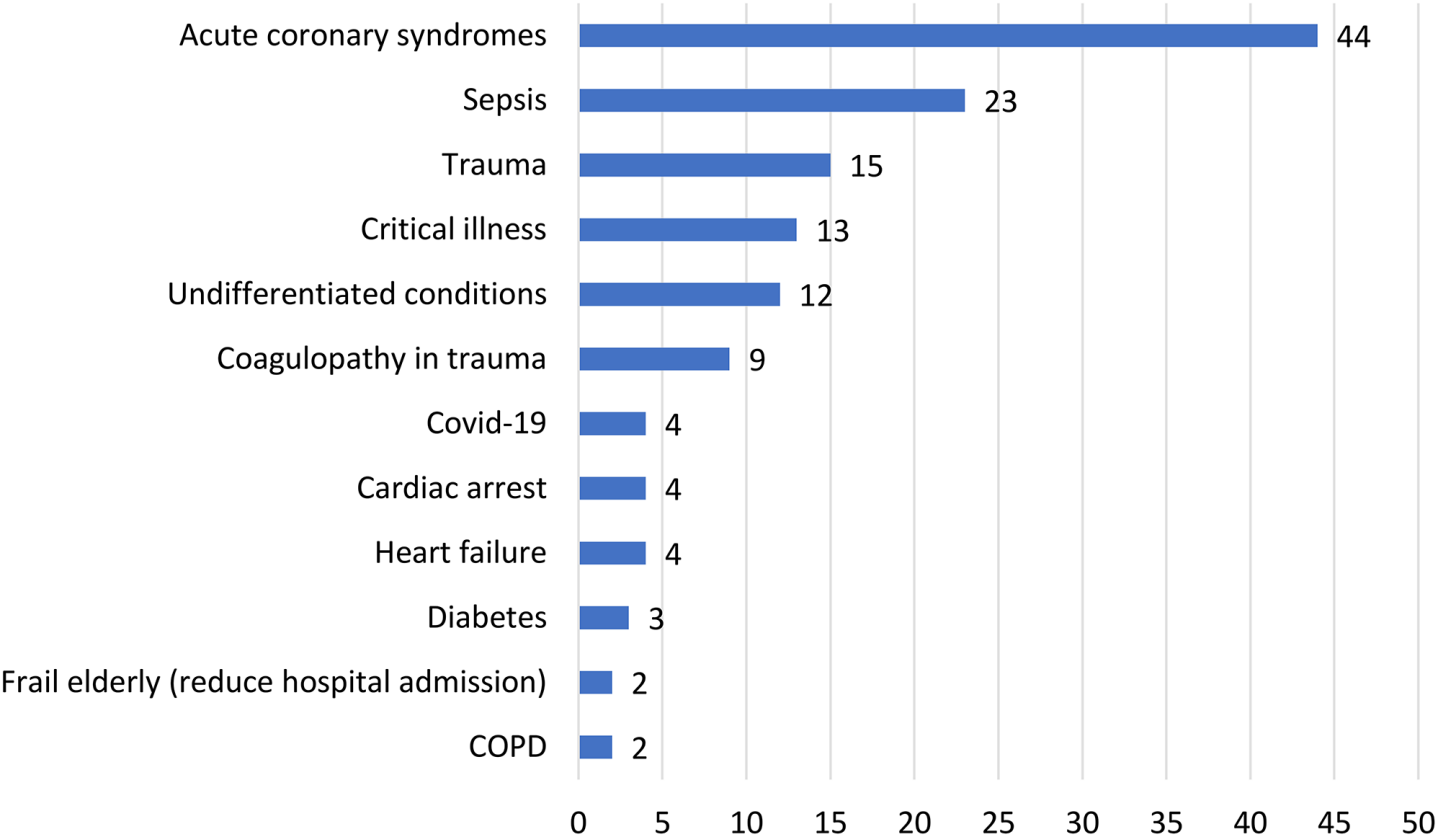
Conditions most frequently reported in research on using POCT in the EMS (*n* = 141 reports). Footnote: COPD Chronic obstructive pulmonary disease. Reports of conditions mentioned fewer than twice are not shown

**Fig. 5:**
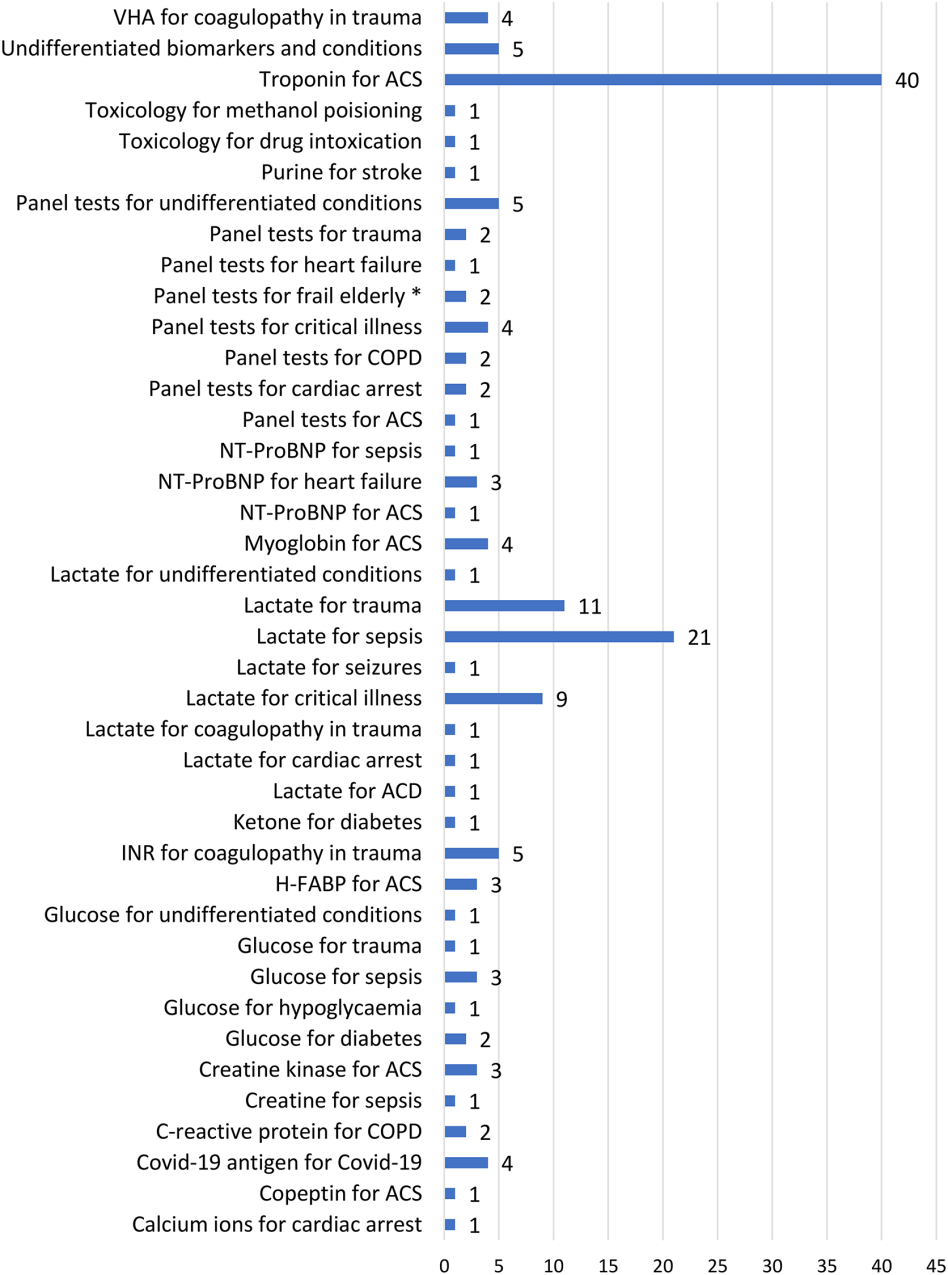
Frequency of *n* = 40 combination of biomarkers and conditions assessed with point-of-care tests in the emergency medical services (*n* = 155). Footnotes: * Frailty in the elderly had the aim to reduce hospital admissions. Denominator is 155 combinations of condition for biomarker as reported in *n* = 141 research reports. ACS Acute cardiovascular syndromes, ACD Acute cardiovascular disease, CK-MB creatine kinase myoglobin, COPD Chronic obstructive pulmonary disease, H-FABP Human-type fatty acid binding protein, INR International Normalized Ratio. Panel tests comprise several biomarker tests done at the same time (see supplementary materials). Undifferentiated conditions and biomarkers refer to papers where the conditions and biomarkers were not specified, VHA Viscoelastic haemostatic assay

The most frequently researched conditions in the included reports were ACS (44/141, 31%), sepsis (23,16%), trauma (15,11%), critical illness (13,9%) and coagulopathy in trauma (9,6%) (Fig. 4). We looked at combinations of biomarkers and conditions of which there were 40 types (Fig. 5). The 141 papers reported a total of 155 combinations of biomarker and condition. Most (94%,133) reported a single biomarker condition combination, with 8 reporting between 2 and 4 combinations (8,6%). The most frequently researched combination of biomarker and condition was troponin for.

ACS (41,27%). Lactate was researched for sepsis (22,14%), for trauma (11,7%), and for critical illness (9,6%) (Fig. 5; Table 1). Other biomarkers researched for ACS in the EMS were creatine kinase, copeptin, H-FABP (heart-type fatty acid binding protein), myoglobin, NT-ProBNP (N-terminal Beta-Natriuretic Peptide), and panel tests (13,8%). Also relatively frequently researched for trauma, in diagnostic test accuracy (DTA) studies or in early, developmental research, were Internationalized normal ratio (INR) (5,4%) and visco-haemoelastic assay (VHA) (4,3%). Panel tests were frequently researched for: critical illness (4,3%); feasibility of use of panel test equipment in an undifferentiated population (5,3%); chronic obstructive pulmonary disease (COPD) (4,3%); trauma (2,1%), cardiac arrest (2,1%); reducing hospital admission in the frail elderly (2,1%); and heart failure (1,1%) (Fig. 4; Table 2).

Conditions assessed in the 21/141 reports set in HEMS/fixed-wing setting were focussed towards trauma (8,38%), critical illness (3,14%) sepsis (2, 10%), cardiac arrest (1,5%) and heart failure (1,5%). Tests in HEMS included viscoelastic assays, INR or prothrombin time and lactate, panel tests for electrolytes, haematology, blood gases and chemistry. These were mostly investigated using DTA studies and developmental type studies investigating the suitability of using the test devices while flying. None of the studies in HEMS or fixed wing were designed to assess changes in the care pathway or associated patient outcome.

### Test devices and manufacturers

**Fig. 6:**
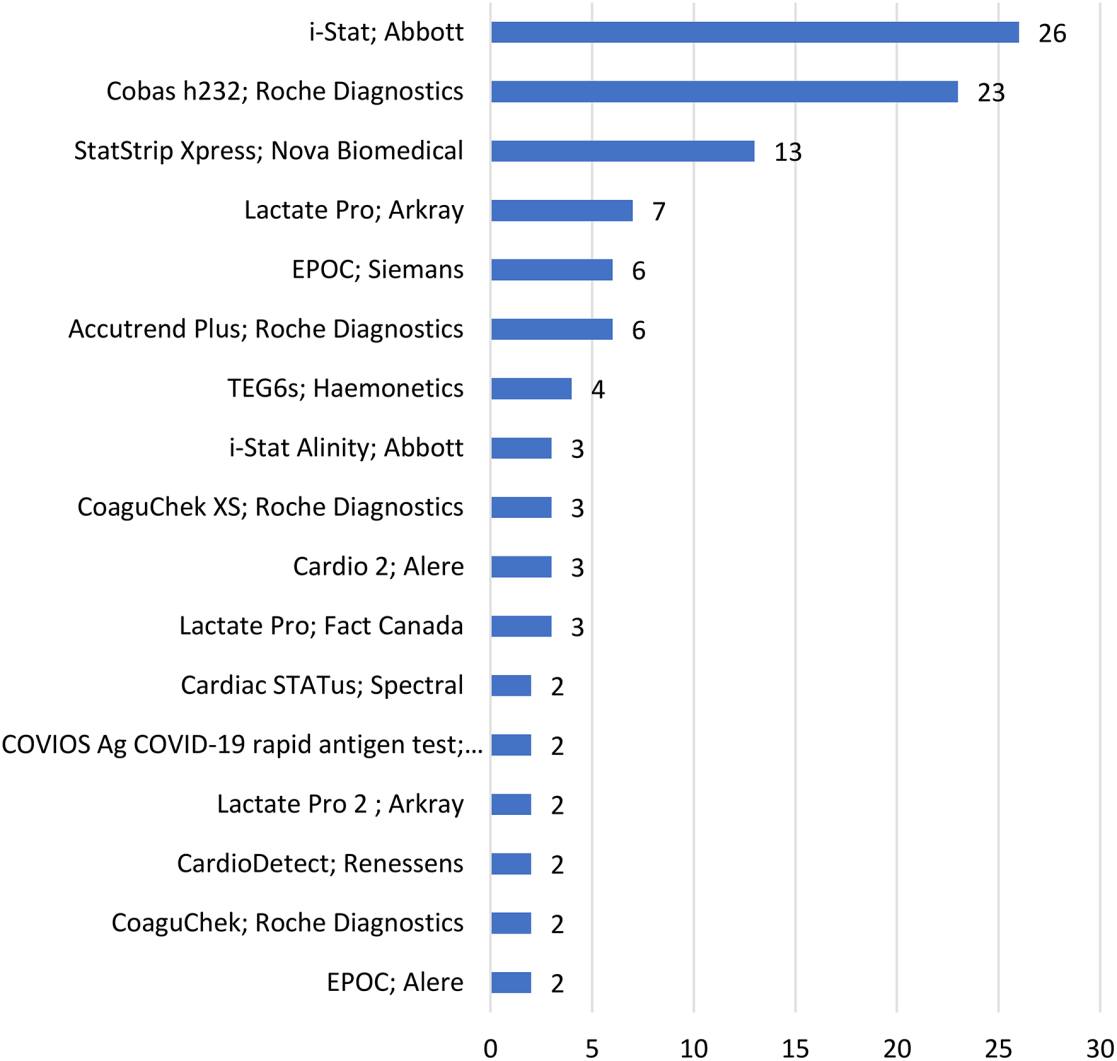
Number of the eleven most frequently reported point-of-care test devices *n* = 161

Most papers (*n* = 141) (120,85%) specified the manufacturer and model of the (POCT) device used but 21 (15%) did not report the name or manufacturer clearly. Many reports included more than one POCT device and the denominator for POCT devices was *n* = 161. Most frequently reported POCT devices were the I-Stat from Abbott Laboratories (26,16%), the Cobas h232 by Roche Diagnostics (23,14%) and the StatStrip Xpress, by Nova Biomedical (13,8%) (Fig. 5B). A total of 41 different devices were listed across the research reports. The most frequently researched devices (11,7%) were referred to between 3 and 26 times, with a significant “tail” including devices mentioned only once (22,14%) or twice (6,4%) Fig. 6 (See supplemental material).

### Acceptability of POCT to patients and staff, from qualitative research and surveys

Four research reports (4,3%) undertook qualitative research. One asked patients for the views on POCT for COPD in the EMS, two looked at acceptability of Covid-19 testing in the EMS and one (a protocol) asked EMS staff about the feasibility of using any type of POCT in the EMS [55–58]. Nine research reports (9/158, 6%) included a survey of use of POCT in the EMS. Four surveyed EMS staff and one surveyed patients [55, 59–66]. Four of these were investigating the adoption of POCT in the EMS across EMS services for a variety of use cases. For example, one survey of 104 EMS regions across 18 countries in Europe for protocols for heart failure indicated that fewer than 25% carried POCT capability and that 6% had POCT for troponin and 3.5% had POCT for NT-ProBNP [61]. A survey of air ambulance services in the UK reported 6/22 services carried POCTs for trauma evaluation [65]. A survey of diabetes provision in EMS in Germany indicated that carriage of ketone strips for POCTs dwindled between 2002 and 2022, as a result of evidence based protocols related to treating suspected hyperglycaemia [63, 64]. It should be noted that apparent effects of introducing POCTs in these examples have not been formally tested in RCTs or NRSI, but do illustrate that POCTs are being used in various EMS.

## Discussion

We have described research in POCT in the EMS published in the last 23 years. The evidence base is dominated by assessments of diagnostic accuracy with very few investigating the effects of the introduction of POCT on the patient pathway and associated outcomes, economic analyses or the opinions of patients or carers or emergency medical staff on the use of POCT in the EMS. The most frequently researched combination was POCT troponin for ACS. Also frequently researched was POCT lactate for sepsis, lactate for trauma and/or lactate for critical care. It was notable that despite identifying 141 full-text research reports including 158 study designs and 155 combinations of biomarker and condition, only troponin and lactate had been evaluated in either RCTs or NRSI to assess the effect of the POCT on the patient pathway and associated outcomes. There was no qualitative research on either POCT troponin or lactate use. The variation between the quantitative and qualitative research on POCT highlights a mismatch between the POCTs researched most frequently (troponin and lactate) and lack of research designed to assess health outcomes. Over half of the research was conducted in three countries, The Netherlands, The US and the UK. research was in the.

Acceptability of the use of POCTs in EMS using qualitative research methods was reported in only four reports and the focus was POCTs for Covid-19 or COPD, with one paper examining thoughts on the use of POCT in EMS in general [55–58]. Although several reports of satisfaction surveys of patients and EMS staff were identified, surveys might be subject to responder bias, e.g. patients or carers of patients who are severely ill may be unable to complete a questionnaire or respondents may feel the need to respond positively to please the researcher [67]. The surveys of POCT availability on the EMS indicated a limited introduction of new tests. A systematic review of barriers and facilitators of the acceptability of POCT in the hospital setting reported that 35% of papers mentioned reduced staff satisfaction and increased friction between staff groups (clinical staff and laboratory staff) [2]. Lack of qualitative research might be an artefact of little formal implementation research of new POCT in the EMS, the stage at which qualitative research might often be undertaken. This emphasizes the need for qualitative research to explore the views and experiences of EMS staff, patients and hospital clinicians on the use of POCT in the EMS.

The lack of essential economic information for policy makers to implement POCT was highlighted a decade ago along with a lack of assessments of effectiveness in relation to patient outcomes [68, 69]. A review of evidence gaps for POCT in primary care also highlighted that the majority of POCT underwent clinical performance assessment (can the test “diagnose” the condition) but few progressed to evaluation of effects on the patient pathway or cost-effectiveness [25]. This fits with other research showing that RCTs of diagnostic tests that evaluate patient care are rare [70]. We estimate the time between the date of publication between the first clinical performance study (such as diagnostic test evaluation) and the date of publication of the first RCT was 11 years for troponin testing. For sepsis there are not yet any RCTs, and the development time between publication of the first DTA study to the publication of the first NRSI to assess the effects of introducing this POCT into EMS was 6 years. These timelines are similar to the time to evaluation for POCTs in primary care which has a median duration of 9 years (Interquartile range 6 to 13 years) [25]. The cycle of evaluation of POCTs is not as clear cut as for other interventions in healthcare, and it is reported that POCTs are sometimes rolled out into service without evidence of clinical effectiveness [25, 71]. Delays in the progression to evaluation for POCTs might be a reflection of the difficulties of introducing POCTs into established healthcare settings [2]. For example the KARMA2 RCT (published after our search date) indicated that they had a low recruitment rate, and cites the effect of the COVID 19 pandemic on capacity of staff and service to engage in research activities and recommended other research designs, such as cluster RCT [72].

Our findings are reflected in the systematic reviews of POCTs that we identified [6–12, 17, 18].These reviews similarly included data from the RCTs, NRSI and diagnostic test accuracy studies but they often applied broader eligibility criteria to encompass settings other than the EMS, such as intensive care, emergency departments and primary care. This may reduce generalizability as the populations differ from those requiring an EMS response.

We used a broad search strategy run across three electronic bibliographic databases (MEDLINE, Embase and CINAHL) to help minimize the risk of missing key studies. We ran supplementary searches for specific biomarkers considered to be the most likely candidates for POCTs in EMS and these will have undoubtedly increased our identification of evidence for certain POCTs. The choice of biomarkers for supplementary searching will have been informed by our stakeholders personal and professional research interests and experiences of researching, working in or using emergency medical services. It should be noted that due to time constraints, the initial focus of this research project was to support a grant application for a platform trial [32], the searches favoured specificity over sensitivity. Also, we did not run a separate search for grey literature, which would have included the international registers, where additional RCTs may have been registered. We followed established guidelines for conducting systematic scoping reviews, enhancing transparency and reproducibility. This included clearly defined inclusion criteria and a rigorous screening process. We used systematic data extraction, with multiple reviewers involved to ensure accuracy and reliability. We have presented our findings in a structured report, with descriptive summaries to facilitate the identification of key themes and gaps in the existing literature following PRISMA guidance [30]. The search was not designed to investigate the very early technical development of POCTs for EMS, which we categorized as “Development” for example shaking the POCT device to mimic the effect of a moving vehicle. To investigate this early developmental phase would potentially need specific search terms, bibliographic, databases and research methods such as horizon scanning, or contact with manufacturers.

This scoping review has identified considerable gaps in the current research literature and a tension between what has been achieved in research and what is needed. Firstly, that there is slow research progress from initial assessments of POCT to the commissioning and publication of RCTs, qualitative work on acceptability and economic analyses. Secondly, that to implement new strategies in the EMS robust evidence on causality and economics are needed, and thirdly, there is very little evidence of a complete assessment other than the use of POCT troponin for acute coronary syndromes. The reasons for lack of a complete research pipeline can be inferred, that RCTs of tests are challenging to conduct [73, 74], are expensive and slow to set up and to report [70], that qualitative work is frequently done in conjunction with implementation or nested within RCTs and there is very little implementation of new POCTs in the EMS. Design recommendations for clinical evaluation of diagnostic tests within health-care services is described in the Medical Services Advisory Committee guidelines (MSAC) [75]. RCTs can deliver the most direct evidence, from “actual management” by comparing services with and without the test, but the most common design for change in management are observational “before and after” studies which can be used if considerations about the purpose of the test and potential biases in “before and after” designs are considered [75].

## Recommendations

Based on our findings we recommend the following:

- High quality randomised controlled trials of the clinical, cost effectiveness and system-wide impact of the most promising POCTs in EMS. If RCTs are not feasible, then robust observational designs taking into account potential biases could be considered.
- Wherever possible, these trials should consider the impact of POCTs in EMS on the patient pathway and patient-centred outcomes such as transport to hospital, need for further care and safety (as described in Lijmer et al. and Verbakel et al.) [25, 71].
- Efforts to reduce the time between initial diagnostic accuracy evaluations of POCT in EMS and their evaluation in high-quality clinical trials.
- Examination of the acceptability of POCT in the EMS using qualitative research with patients, their carers, EMS and other staff.
- Systematic reviews should adjust inclusion criteria to focus on the EMS setting, and a population requiring emergency medical transport, or present results stratified by setting and/or population.

## Conclusions

We have identified that the research landscape for POCT in EMS is active, and the amount of research has increased substantially in the last 10 years. Research is focussed on a few, well-known POCT candidates (troponin for ACS and lactate for sepsis and trauma) with a long tail of different biomarkers and conditions investigated in one or two research papers. Most research is aimed at clinical performance assessment, by establishing diagnostic accuracy, but a small number of POCTs are progressing to formal evaluation of their clinical effectiveness, cost effectiveness and acceptability. Use of RCTs to evaluate the effectiveness is ideal but may be hard to achieve, therefore robust observational designs, for example as recommended by MSAC, could be considered. Attempts to synthesise the evidence in systematic reviews are affected by the paucity of primary research and inclusion of settings/populations other than the EMS. There is considerable opportunity for further research to guide this growing area of clinical practice.

**Table 1:** Comprehensive list of condition and biomarker combinations (*n* = 155) reported in 141 full-text research reports

Biomarkers	Calcium ions	Copeptin	Covid 19 antigen	Creatine kinase	Creatinine	H-FABP	Glucose	INR	Ketone	Lactate	Myoglobin	NT Pro BNP	Panel Tests	Purine	Toxicology	Troponin	VHA	Undifferentiated bio-markers	Grand total
Conditions	n(%)		n(%)			n(%)	n(%)	n(%)	n(%)	n(%)		n(%)	n(%)	n(%)	n(%)	n(%)	n(%)	n(%)	n(%)
ACD										1(0.6)									1 (0.6)
ACS		1(0.6)		1(0.6)		3(1.8)					4 (2.6)	1(0.6)				41(27.2)			53 (34.1)
Cardiac arrest	1(0.6)									1(0.6)			2(1.3)						4 (2.6)
Coagulopathy in trauma								4(2.6)		1(0.6)							4(2.6)		9(5.8)
COPD													4(2.6)						4 (2.6)
Covid-19			4(2.6)																4 (2.6)
Critical illness										9(5.8)			4(2.6)						13 (8.4)
Diabetes							2(1.3)		1(0.6)										3 (1.9)
Drug intoxication															1(0.6)				1 (0.6)
Frail elderly *													2(1.3)						2 (1.3)
Heart failure												3(2.1)	1(0.6)						4 (2.6)
Hypoglycaemia							1(0.6)												1 (0.6)
Methanol poisoning															1(0.6)				1 (0.6)
Seizures										1(0.6)									1 (0.6)
Sepsis					1(0.6)		2(1.3)			22(14.1)		1(0.6)							26 (16.7)
Stroke														1(0.6)					1 (0.6)
Trauma							1(0.6)	1(0.6)		11(7.1)			2(1.3)						15 (9.7)
Undifferentiated conditions							1(0.6)			1(0.6)			5(3.2)					5(3.2)	12 (7.7)
Grand Total	1(0.6)	1(0.6)	4(2.6)	1(0.6)	1(0.6)	3(1.8)	6(3.8)	5(3.2)	1(0.6)	47(31.1)	4 (2.6)	5(3.2)	20(12.9)	1(0.6)	2(1.3)	41(27.2)	4(2.6)	5(3.2)	155 (100)

Footnote: *Frail elderly (aim was to prevent unnecessary admissions to hospital. ACS Acute coronary syndromes. ACD Acute cardiovascular disease. COPD Chronic obstructive pulmonary disease. H-FABP Human-type fatty acid binding protein. NT-ProBNP N-terminal Beta-Natriuretic Peptide. Undifferentiated conditions and biomarkers refer to papers where the conditions and biomarkers were not specified

**Table 2:** Research study design for combined mentions of point-of-care tests for a biomarker paired with a specific condition (*n* = 155), as identified from *n* = 141 research reports

Study designs Biomarker for conditions	Systematic review	RCT	Economic evaluation	NRSI (Comparative cohort study of interventions	Qualitative	Observation	Survey	DTA	Development	Grand Total
	*n*(%)	*n*(%)	*n*(%)	*n*(%)	*n*(%)	*n*(%)	*n*(%)	*n*(%)	*n*(%)	*n*(%)
Calcium ions for cardiac arrest							1(0.6)			1(0.6)
Copeptin for ACS								1(0.6)		1(0.6)
Covid-19 antigen for Covid-19					1(0.6)			2(1.3)		4(2.6)
Creatine for sepsis								1(0.6)		1(0.6)
Creatine kinase for ACS								1(0.6)		3(1.9)
C-reactive protein for COPD					1(0.6)	1(0.6)				2(1.3)
H-FABP for ACS	2(1.3)							1(0.6)		3(1.9)
Glucose for diabetes					1(0.6)		1(0.6)			2(1.3)
Glucose for hypoglycaemia									1(0.6)	1(0.6)
Glucose for sepsis								2(1.3)		2(1.3)
Glucose for trauma						1(0.6)				1(0.6)
Glucose for undifferentiated conditions						1(0.6)				1(0.6)
INR for coagulopathy in trauma	1(0.6)						1(0.6)	3(1.9)	1(0.6)	6(3.1)
Ketone for diabetes							1(0.6)			1(0.6)
Lactate for ACD								1(0.6)		1(0.6)
Lactate for cardiac arrest								1(0.6)		1(0.6)
Lactate for coagulopathy in trauma								1(0.6)		1(0.6)
Lactate for critical illness	1(0.6)							8(5.0)		9(6.0)
Lactate for seizures								1(0.6)		1(0.6)
Lactate for sepsis	4(2.6)			1(0.6)			1(0.6)	13(8.4)	2(1.2)	21(14.0)
Lactate for trauma	4(2.6)							6(4.0)	1(0.6)	11(7.3)
Lactate for undifferentiated conditions									1(0.6)	1(0.6)
Myoglobin for ACS								4(2.6)		4(2.6)
NT-proBNP for ACS								1(0.6)		1(0.6)
NT-proBNP for heart failure								2(1.2)	1(0.6)	3(1.9)
NT-proBNP for sepsis								1(0.6)		1(0.6)
Panel tests for ACS								1(0.6)		1(0.6)
Panel tests for cardiac arrest						2(1.3)				2(1.3)
Panel tests for COPD					1(0.6)		1(0.6)			2(1.3)
Panel tests for critical illness						3(1.9)			1(0.6)	4(2.6)
Panel tests for frail elderly *						2(1.3)				2(1.3)
Panel tests for heart failure							1(0.6)			1(0.6)
Panel tests for trauma						1(0.6)		1(0.6)		2(1.3)
Panel tests for undifferentiated conditions					1(0.6)		1(0.6)	1(0.6)	2(1.2)	5(3.2)
Purine for stroke								1(0.6)		1(0.6)
Toxicology for drug intoxication						1(0.6)				1(0.6)
Toxicology for methanol poisoning									1(0.6)	1(0.6)
Troponin for ACS	5(3.2)	6(4.0)	2(1.3)	8(5.1)		1(0.6)		17(11.0)	1(0.6)	40(26.5)
Undifferentiated biomarkers and conditions	3(1.9)					1(0.6)		1(0.6)		5(3.2)
VHA for coagulopathy in trauma									4(2.6)	4(2.6)
**Grand Total**	**22(14.2)**	**6(3.9)**	**2(1.3)**	**9(5.8)**	**5(3.2)**	**17(11.0)**	**6(3.9)**	**72(46.4)**	**16 (10.3)**	**155(100)**

Footnotes: The denominator in the table represents *n* = 155 instances of combined mentions of point-of-care tests for a biomarker paired with a specific condition, as identified from *n* = 141 research reports. RCT randomised controlled trial. NRSI (Non randomised study of intervention – comparative cohort). DTA diagnostic test accuracy study. Development (experimental study carried out either in controlled conditions e.g. in a laboratory, or using defined samples with known biomarker concentration). Qualitative, qualitative research. ACS Acute coronary syndromes ACD Acute cardiovascular disease COPD Chronic obstructive pulmonary disease, INR International Normalized Ratio, H-FABP Human-type fatty acid binding protein, NT-ProBNP N-terminal Beta-Natriuretic Peptide. VHA Viscoelastic haemostatic assay

## Electronic supplementary material

Below is the link to the electronic supplementary material.

Supplementary Material 1

## Data Availability

The extracted research data are available on Open Science Framework with this identifier: https://doi.org/10.17605/OSF.IO/482BW.
