# Supplementary material for: Point-of-care tests in the emergency medical services: a scoping review
Source: Scand J Trauma Resusc Emerg Med. 2025 Feb 3;33:18. doi: 10.1186/s13049-025-01329-y (PMC11792643; doi:10.1186/s13049-025-01329-y)
Supplement: Supplementary file 1 — Supplementary Material 1 [file 13049_2025_1329_MOESM1_ESM.docx]

# Supplemental material - scoping review

[Supplemental material - scoping review 1](#_Toc175319457)

[Table S1 Searches for point of care tests in the EMS, search terms, dates and number of hits 2](#_Toc175319458)

[Example search strategy 3](#_Toc175319459)

[Table S2 Categorisation of biomarkers assessed using panel tests 6](#_Toc175319460)

[Table S3 Test devices 7](#_Toc175319461)

[List of included studies 10](#_Toc175319462)

[Systematic or systematic scoping review 10](#_Toc175319463)

[Randomised controlled trial 11](#_Toc175319464)

[Economic evaluation 12](#_Toc175319465)

[Non randomised study of intervention (Comparative cohort study) 12](#_Toc175319466)

[Qualitative research 14](#_Toc175319467)

[Surveys 15](#_Toc175319468)

[Diagnostic test accuracy 15](#_Toc175319469)

[Developmental research 20](#_Toc175319470)

# Table S1 Searches for point of care tests in the EMS, search terms, dates and number of hits

|  | **Search** | **Terms** | **Databases** | **Date of search** | **Total *** | **Number of papers summarised for stakeholders** |
| --- | --- | --- | --- | --- | --- | --- |
| **Main search** | | | | | | |
| 1 | All POC tests in EMS | <POC tests> AND <EMS> | Medline, Embase, CINAHL | Oct 04 2023 | 668 | NA |
| **Supplemental targeted searches** | | | | | | |
| 2 | Troponin for ACS | <POC tests> AND <EMS> <Troponin> | Medline, Embase, CINAHL | Jul 10 2023 | 252 | 36 |
| 3 | NT-Pro-BNP (Naturietic peptide for brain injury or heart Failure) | <POC tests> AND <EMS> <NT-ProBNP> | Medline, Embase, CINAHL | Jul 12 2023 | 188 | 4 |
| 4 | Lactate for trauma or sepsis | <POC tests> AND <EMS> <Lactate> | Medline, Embase, CINAHL | Jul 21 2023 | 457 | 35 (22 Sepsis  14 Trauma, I paper covered both sepsis and trauma) |
| 5 | Beta-HCG (Human Chorionic Gonadotropin) | <POC tests> AND <EMS> <Beta-HCG> | Medline, Embase, CINAHL | Jul 24 2023 | 78 | 0 |
| 6 | Head trauma biomarkers  glial fibrillary acidic protein  (GFAP, S100B, UCH-L1) | <POC tests> AND <EMS> <GFAP> OR < S100B> OR  < UCH-L1 >] | Medline, Embase, CINAHL | Jul 24 2023 | 203 | 0 |
| 7 | Respiratory tract infection (RTI) systematic reviews | <POC tests> AND <EMS> <RTI>  <Systematic review> AND <POC tests> AND <EMS> AND <RTI> | Medline, Embase, CINAHL Cochrane database for systematic reviews | Aug 02 2023 | 244 | 7 |
| 8 | Ketones for diabetic keto-acidosis | <POC tests> AND <EMS> <Ketones> | Medline, Embase, CINAHL | Aug 02 2023 | 195 | 4 |
|  |  |  |  | **Total** | **2285** | **86** |

* Numbers after deduplication and after limiting the search results to the year 2000 or later. All searches were limited to English and focused on adults. Search for RTI = respiratory tract infection included both bacterial and viral. Full search terms available from OSF (*Moore T.H.M., Dawson S., Savovic J., Voss S., Body R., Benger J. Point of Care Test in EMS - Search strategies and search history DOI 10.17605/OSF.IO/8MQ7N. OSF online 2022*). NA = Not applicable; POC = point of care; EMS = Emergency medical services; RTI = respiratory tract infections.

# Example search strategy

Point-of-care testing in the emergency medical services First search.

MEDLINE, CINAHL Date-of-search: 18-Aug-2022

Search-2 strategy

Ovid Multifile Search

Embase <1974 to 2022 October 04>

Ovid MEDLINE(R) ALL <1946 to October 04, 2022>

[Intervention-1: POCT – generic search terms]

1 "point of care system"/ or point-of-care systems/

2 "point of care testing"/ or rapid on-site evaluation/

3 POCT?.mp.

4 (((point adj2 care) or poc) adj3 (analys* or assay* or immunoassay* or classif* or detect* or diagnos* or differenti* or method* or predict* or rapid or routine* or system* or technique* or test or tests or testing)).ti,ab,kf.

5 (((point adj2 care) or poc) adj3 (devic* or platform? or panel?)).ti,ab,kf.

6 ((invitro or in-vitro) adj2 (diagnos* or test or tests or testing or device*)).ti,ab,kf.

7 (rapid adj2 (screen* or test or tests or testing)).ti,ab,kf.

8 (near patient? adj3 (analys* or assay* or immunoassay* or classif* or detect* or diagnos* or differenti* or method* or predict* or technique* or test or tests or testing)).ti,ab,kf.

9 (((mobile or portable or handheld or hand-held) adj3 (analy?er? or device? or meters or metres)) and (blood? or plasma or saliva or sputum or spit or mucus or urine or urea or urinalys* or fluids or gas or gases)).mp.

10 or/1-9

[Setting]

11 ambulance/ or ambulances/ or ambulance transportation/

12 air ambulances/ or air medical transport/

13 emergency responders/ or "first responder (person)"/ or rescue personnel/

14 paramedical personnel/ or emergency medical technicians/

15 emergency medical dispatch/

16 ambulanc*.mp.

17 ((ambulatory or mobile) adj emergenc*).ti,ab,kf.

18 ((helicopter? and emergenc*) or aeromedic* or aero-medic* or airmedic* or air medic*).ti,ab,kf.

19 (emergenc* adj2 (craft? or crew or dispatch* or personnel or responder? or technician? or transport* or vehicle? or helicopter?)).ti,ab,kf.

20 (paramed* or para-med*).ti,ab,kf.

21 ((first or frontline or front line) adj responder?).ti,ab,kf.

22 or/11-21

23 (10 and 22)

24 *Emergency Medical Services/ and ("point of care testing"/ or rapid on-site evaluation/)

25 exp Emergency Medical Services/ and ("point of care testing"/ or rapid on-site evaluation/) and (prehospital* or pre-hospital* or out-of-hospital*).mp.

26 or/23-25

[Intervention-2: PoCT – analytes commonly tested]

27 ((Point-of-care or POC or POCT? or triage) adj3 (blood? or bicarb* or calcium or chloride or creatinine or d-dimer? or glucose or h?emoglobin or h?ematocrit or lactate or lactic or myoglobin or peptide? or BNP or NT-proBNP or potassium or prothrombin or INR or sodium or troponin or TnT or cTnI)).ti,ab,kf.

28 ((Point-of-care or POC or POCT? or triage) adj3 (clot* or coagulat* or anticoagulat* or thrombo*)).ti,ab,kf.

[Intervention-3: Named POCT Tests identified from Search-1]

29 Accutrend Plus*.af.

30 AQT90 Flex*.af.

31 Atellica VTLi*.af.

32 (Arterial Blood Gas adj3 Kit?).af.

33 ((blood gas analy* or radiomet*) and ("ABL-90" or ABL90* or ABL-90TM or ABL90TM)).af.

34 (CARDIAC Trop T Sensitive or Cardiac Reader).af.

35 ((Cardio2 or Cardio-2 or Cardio3 or Cardio-3) and cTnI).af.

36 Cardio? panel.af.

37 (Chem-7* or Chem7*).af.

38 Chemstrip* bg*.af.

39 CoaguChek*.af.

40 ("cobas h232*" or "cobas h 232*" or "cobash232*").af.

41 ((epoc?? and (Alere or Siemens)) or (epoc?? adj3 (POC?? or test? or device? or blood analysis system?))).af. or EPOC??.mv,my,dm,dv.

42 ExacTech*.af.

43 (i-stat or i-statTM).af. or i-stat*.mv,my,dm,dv. or (i-STAT* and (CG4? or EC8?)).af.

44 Labkit*.af.

45 (Lactate adj ("Pro-2" or "Pro2")).af.

46 LifeSign*.af.

47 HemoCue*.af.

48 PU-4010*.af.

49 (Menarini* and Aution* and Micro*).af.

50 pocketchem*.af.

51 ("Rad-57" or "Rad57" or Rad-57TM or Rad57TM).af.

52 ((radiomet* or blood gas analy*) and ("ABL-90" or ABL90* or ABL-90TM or ABL90TM)).af.

53 ((SARS-CoV-2 or SARS-CoV2 or COVID* or Coronavir*) adj3 ((ag or antigen?) adj3 (detect* or diagnos* or screen* or test or tests or testing))).af.

54 (((SARS-CoV-2 or SARS-CoV2 or COVID* or Coronavir*) adj7 (detect* or diagnos* or screen* or test or tests or testing)) and (Abbot or CLINITEST or GenBody or GENEDIA or Healgen or Helix or Humasis or Roche or Vazyme or VivaDiag)).af.

55 SMARTChip*.af.

56 StatStrip*.af.

57 (Stratus CS or Stratus CSTM).af. or Stratus CS*.mv,my,dm,dv.

58 ("TEG 6s" or "TEG6s" or "TEG6 s" or (ROTEM* adj3 sigma)).af.

59 (Triage MeterPlus* or (Triage adj (meter* or metre*))).af.

60 Triage MeterPro*.af.

61 or/27-60

62 (22 and 61)

63 (prehospital* or pre-hospital* or out-of-hospital).ti,kf.

64 (61 and 63)

65 (26 or 62 or 64)

[Limits]

66 ((infant* or child* or p?ediatri*) not adult*).ti.

67 65 not 66

68 limit 67 to conference abstract status [Embase records only, n=179]

69 67 not 68

70 limit 69 to english language

*******************************************************************

# Table S2 Categorisation of biomarkers assessed using panel tests

| **Grouped as** |  |
| --- | --- |
| **Panel test name** | **Biomarkers evaluated** |
| Electrolytes | Sodium ions (Na+) |
|  | Potassium ions (K+) |
|  | Chloride (Cl-) |
|  | TCO2 (total Carbon dioxide) |
|  | Anion Gap* |
|  | Calcium ions (Ca++) |
|  | Glucose (Glu) |
|  | Urea Nitrogen (BUN)/Urea |
|  | Creatinine (Crea) |
| Haematology | Haematocrit (Hct) |
|  | Haemoglobin* (Hgb) |
| Blood gases | pH |
|  | PCO2 (partial pressure carbon dioxide) |
|  | PO2 (partial pressure carbon dioxide) |
|  | TCO2* (total carbon dioxide) |
|  | HCO3* (Bicarbonate) |
|  | BE (Base Excess)* |
|  | sO2 (Saturation of O2) |
| Chemistry | Lactate |
| Viscoelastic haemostatic assays | (R time, K time, Alpha-angle slope and Maximum amplitude) |
| INR | International normalized ratio |

*If a study measured lactate on its own, it would be categorized as a single biomarker however if the study measured lactate with numerous other panel test, and the focus of the study was not specifically on lactate, it would be classified as a panel test for chemistry.

# Table S3 Test devices

List of point-of-care test devices and manufacturers (n=161) mentioned in research reports (from n=141 full text papers).

| **N** | **Test device and manufacturer** | **Count** |
| --- | --- | --- |
| 1 | i-Stat; Abbott | 26 |
| 2 | Cobas h232; Roche Diagnostics | 23 |
| 3 | StatStrip Xpress; Nova Biomedical | 13 |
| 4 | Lactate Pro; Arkray | 7 |
| 5 | Accutrend Plus; Roche Diagnostics | 6 |
| 6 | EPOC; Siemans | 6 |
| 7 | TEG6s; Haemonetics | 4 |
| 8 | Lactate Pro; Fact Canada | 3 |
| 9 | Cardio 2; Alere | 3 |
| 10 | CoaguChek XS; Roche Diagnostics | 3 |
| 11 | i-Stat Alinity; Abbott | 3 |
| 12 | CardioDetect; Renessens | 2 |
| 13 | EPOC; Alere | 2 |
| 14 | COVIOS Ag COVID-19 rapid antigen test; Mologic | 2 |
| 15 | Lactate Pro 2 ; Arkray | 2 |
| 16 | CoaguChek; Roche Diagnostics | 2 |
| 17 | Cardiac STATus; Spectral | 2 |
| 18 | Rapicheck; Dainippon pharmaceutical | 1 |
| 19 | Experimental portable breath detector; NR | 1 |
| 20 | Lateral flow devices; various | 1 |
| 21 | Trop T; Roche | 1 |
| 22 | Optimum Xceed; Abbott | 1 |
| 23 | Accutrend Lactate Meter; Roche Diagnostics | 1 |
| 24 | SMARTCHIP; Not reported | 1 |
| 25 | Drugwipe 6s; Securetec Detektions-Systeme | 1 |
| 26 | Lifepak 12; Mediatronic Physiocontrol | 1 |
| 27 | LABGEO; Samsung | 1 |
| 28 | Cardiac reader; Roche Diagnostics | 1 |
| 29 | Lactate Plus; Nova Biomedical | 1 |
| 30 | QuickRead Go; Brondby Denmark | 1 |
| 31 | Contour TS; Contour | 1 |
| 32 | ROTEM-Sigma; Werfen | 1 |
| 33 | EPOC; Epocal inc Canada | 1 |
| 34 | Triage MeterPlus; Biosite SAS | 1 |
| 35 | Accusport; Boehringer PLC, Ingelheim, Germany | 1 |
| 36 | Fiber optic particle plasmon resonance; in development | 1 |
| 39 | Triage; Biomedical diagnostics, France | 1 |
| 37 | Glucotouch; LifeScan Inc | 1 |
| 38 | ABL-90; Radiometer | 1 |
| 39 | HemoCue point of care test; HemoCue | 1 |
| 40 | Not reported Either no name was reported a full name and manufacturer was not reported. | 27 |
| 41 | Many* | 3 |
|  | Grand Total | 161 |

*Three papers reported more than 10 devices

# List of included studies – organized by study type

Systematic or systematic scoping review

ALGHAMDI, A., ALOTAIBI, A., ALHARBI, M., REYNARD, C. & BODY, R. 2020. Diagnostic Performance of Prehospital Point-of-Care Troponin Tests to Rule Out Acute Myocardial Infarction: A Systematic Review. *Prehospital & Disaster Medicine,* 35**,** 567-573.

ALGHAMDI, A. & BODY, R. 2018. BET 1: Prehospital cardiac troponin testing to 'rule out' acute coronary syndromes using point of care assays. *Emergency Medicine Journal,* 35**,** 572-574.

BRUINS SLOT, M. H. E., REITSMA, J. B., RUTTEN, F. H., HOES, A. W. & VAN DER HEIJDEN, G. J. M. G. 2010. Heart-type fatty acid-binding protein in the early diagnosis of acute myocardial infarction: A systematic review and meta-analysis. *Heart,* 96**,** 1957-1963.

DEMANDT, J. P. A., ZELIS, J. M., KOKS, A., SMITS, G., VAN DER HARST, P., TONINO, P. A. L., DEKKER, L. R. C., VAN HET VEER, M. & VLAAR, P. J. 2022. Prehospital risk assessment in patients suspected of non-ST-segment elevation acute coronary syndrome: a systematic review and meta-analysis. *BMJ Open,* 12**,** e057305.

FUZERY, A. K., ELIAN, F. A. & KOST, G. J. 2022. A review of temperature-related challenges and solutions for the Abbott i-STAT and Siemens Healthineers epoc devices. *Clinical Biochemistry,* 05**,** 05.

GOYDER, C., TAN, P. S., VERBAKEL, J., ANANTHAKUMAR, T., LEE, J. J., HAYWARD, G., TURNER, P. J. & VAN DEN BRUEL, A. 2020. Impact of point-of-care panel tests in ambulatory care: a systematic review and meta-analysis. *BMJ Open,* 10**,** e032132.

GREENE, J., GOLDSTEIN, J. P., LANE, D. J., JENSEN, J. L., LEROUX, Y., SWAIN, J., FIDGEN, D., BROWN, R., SIMPSON, M. & CARTER, A. J. E. 2021. State of the evidence for emergency medical services care of adult patients with sepsis: An analysis of research from the prehospital evidence-based practice program. *Australasian Journal of Paramedicine,* 18**,** 1-6.

HO, C., CIMON, K., JABR, M., MIERZWINSKI-URBAN, M., CLEMENT, F., SORIL, L., DUNFIELD, L., WEEKS, L. & REYNARD, P. 2015. Point-of-Care Cardiac Troponin Testing in Patients With Symptoms Suggestive of Acute Coronary Syndrome - Project Protocol. *Canadian Agency for Drugs and Technologies in Health. CADTH Optimal Use Reports,* 06**,** 06.

HO, C., CIMON, K., JABR, M., MIERZWINSKI-URBAN, M., CLEMENT, F., SORIL, L., DUNFIELD, L., WEEKS, L. & REYNARD, P. 2016a. Point-of-Care Troponin Testing in Patients With Symptoms Suggestive of Acute Coronary Syndrome: recommendations. *Canadian Agency for Drugs and Technologies in Health. CADTH Optimal Use Reports*.

HO, C., CIMON, K., JABR, M., MIERZWINSKI-URBAN, M., CLEMENT, F., SORIL, L., DUNFIELD, L., WEEKS, L. & REYNARD, P. 2016b. Point-of-Care Troponin Testing in Patients With Symptoms Suggestive of Acute Coronary Syndrome: Health Technology Assessment. *Canadian Agency for Drugs and Technologies in Health. CADTH Optimal Use Reports*.

HO, C., CIMON, K., JABR, M., MIERZWINSKI-URBAN, M., CLEMENT, F., SORIL, L., DUNFIELD, L., WEEKS, L. & REYNARD, P. 2016c. Point-of-Care Troponin Testing in Patients With Symptoms Suggestive of Acute Coronary Syndrome: In Brief. *Canadian Agency for Drugs and Technologies in Health. CADTH Optimal Use Reports*.

JONES, C. & LIGHTOWLER, B. 2022. The efficacy of the HEART score in prehospital settings. *Journal of Paramedic Practice,* 14**,** 198-211.

KIRBY, K. 2013. Pre-hospital lactate testing in the identification of patients with sepsis: a review of the literature. *Journal of Paramedic Practice,* 5**,** 698-703.

KRUSE, O., GRUNNET, N. & BARFOD, C. 2011. Blood lactate as a predictor for in-hospital mortality in patients admitted acutely to hospital: a systematic review. *Scandinavian journal of trauma, resuscitation and emergency medicine,* 19**,** 74.

LEWIS, C. T., NAUMANN, D. N., CROMBIE, N. & MIDWINTER, M. J. 2016. Prehospital point-of-care lactate following trauma: A systematic review. *The Journal of Trauma and Acute Care Surgery,* 81**,** 748-55.

MCCLELLAND, G., YOUNGER, P. & BYERS, S. 2012. Lactate measurement in pre-hospital care: a review of the literature. *Journal of Paramedic Practice,* 4**,** 329-334.

MORTON, S., AVERY, P., PAYNE, J. & M, O. M. 2022. Arterial Blood Gases and Arterial Lines in the Prehospital Setting: A Systematic Literature Review and Survey of Current United Kingdom Helicopter Emergency Medical Services. *Air Medical Journal,* 41(2)**,** 201-208.

NEWGARD, C. D., CHENEY, T. P., CHOU, R., FU, R., DAYA, M. R., O'NEIL, M. E., WASSON, N., HART, E. L. & TOTTEN, A. M. 2020. Out-of-hospital Circulatory Measures to Identify Patients With Serious Injury: A Systematic Review. *Academic emergency medicine : official journal of the Society for Academic Emergency Medicine,* 27**,** 1323-1339.

ROBINSON, S. 2019. Is prehospital lactate testing useful in improving clinical assessment? *Journal of Paramedic Practice,* 11**,** 246-253.

ROBINSON, S. & KIRTON, J. 2020. Tools to predict acute traumatic coagulopathy in the pre-hospital setting: a review of the literature. *British Paramedic Journal,* 5**,** 23-30.

SMYTH, M. A., BRACE-MCDONNELL, S. J. & PERKINS, G. D. 2016. Identification of adults with sepsis in the prehospital environment: A systematic review. *BMJ Open,* 6**,** e011218.

STOJEK, L., BIELER, D., NEUBERT, A., AHNERT, T. & IMACH, S. 2023. The potential of point-of-care diagnostics to optimise prehospital trauma triage: a systematic review of literature. *European journal of trauma and emergency surgery : official publication of the European Trauma Society*.

TOTTEN, A. M., CHENEY, T. P., O'NEIL, M. E., NEWGARD, C. D., DAYA, M., FU, R., WASSON, N., HART, E. L. & CHOU, R. 2018. Physiologic Predictors of Severe Injury: Systematic Review.

Randomised controlled trial

AARTS, G. W. A., CAMARO, C., VAN GEUNS, R. J., CRAMER, E., VAN KIMMENADE, R. R. J., DAMMAN, P., VAN GRUNSVEN, P. M., ADANG, E., GIESEN, P., RUTTEN, M., OUWENDIJK, O., GOMES, M. E. R. & VAN ROYEN, N. 2020a. Acute rule-out of non-ST-segment elevation acute coronary syndrome in the (pre)hospital setting by HEART score assessment and a single point-of-care troponin: rationale and design of the ARTICA randomised trial. *BMJ Open,* 10**,** e034403.

AARTS, G. W. A., VAN DER WULP, K. & CAMARO, C. 2020b. Pre-hospital point-of-care troponin measurement: a clinical example of its additional value. *Netherlands Heart Journal,* 28**,** 514-519.

CAMARO, C., AARTS, G. W. A., ADANG, E. M. M., VAN HOUT, R., BROK, G., HOARE, A., RODWELL, L., DE POOTER, F., DE WIT, W., CRAMER, G. E., VAN KIMMENADE, R. R. J., DAMMAN, P., OUWENDIJK, E., RUTTEN, M., ZEGERS, E., VAN GEUNS, R.-J. M., GOMES, M. E. R. & VAN ROYEN, N. 2023. Rule-out of non-ST-segment elevation acute coronary syndrome by a single, pre-hospital troponin measurement: a randomized trial. *European heart journal,* 44**,** 1705-1714.

CAMARO, C., AARTS, G. W. A. & VAN ROYEN, N. 2022. Pre-hospital rule-out of acute coronary syndrome by modified HEART score assessment including point-of-care troponin. *Netherlands heart journal : monthly journal of the Netherlands Society of Cardiology and the Netherlands Heart Foundation,* 30**,** 535.

EZEKOWITZ, J. A., WELSH, R. C., GUBBELS, C., BRASS, N., CHAN, M., KEEBLE, W., KHADOUR, F., KOSHY, T. L., KNAPP, D., SHARMA, S., SOOKRAM, S., TYMCHAK, W., WEISS, D., WESTERHOUT, C. M. & ARMSTRONG, P. W. 2014. Providing Rapid Out of Hospital Acute Cardiovascular Treatment 3 (PROACT-3). *Canadian Journal of Cardiology,* 30**,** 1208-15.

EZEKOWITZ, J. A., WELSH, R. C., WEISS, D., CHAN, M., KEEBLE, W., KHADOUR, F., SHARMA, S., TYMCHAK, W., SOOKRAM, S., BRASS, N., KNAPP, D., KOSHY, T. L., ZHENG, Y. & ARMSTRONG, P. W. 2015. Providing Rapid Out of Hospital Acute Cardiovascular Treatment 4 (PROACT-4). *Journal of the American Heart Association,* 4**,** 01.

Economic evaluation

DAWSON, L. P., NEHME, E., NEHME, Z., ZOMER, E., BLOOM, J., COX, S., ANDERSON, D., STEPHENSON, M., BALL, J., ZHOU, J., LEFKOVITS, J., TAYLOR, A. J., HORRIGAN, M., CHEW, D. P., KAYE, D., CULLEN, L., MIHALOPOULOS, C., SMITH, K. & STUB, D. 2023. Chest Pain Management Using Prehospital Point-of-Care Troponin and Paramedic Risk Assessment. *JAMA internal medicine,* 183**,** 203-211.

VAN DONGEN, D. N., OTTERVANGER, J. P., TOLSMA, R., FOKKERT, M., VAN DER SLUIS, A., VAN 'T HOF, A. W. J., BADINGS, E. & SLINGERLAND, R. J. 2019. In-Hospital Healthcare Utilization, Outcomes, and Costs in Pre-Hospital-Adjudicated Low-Risk Chest-Pain Patients. *Applied health economics and health policy,* 17**,** 875-882.

Non randomised study of intervention (Comparative cohort study)

DEMANDT, J. P. A., KOKS, A., HAEST, R., HEIJMEN, E., THIJSSEN, E., OTTERSPOOR, L. C., VAN VEGHEL, D., EL FARISSI, M., EERDEKENS, R., VERVAAT, F., PIJLS, N. H. J., VEER, M. V. T., TONINO, P. A. L., DEKKER, L. R. C. & VLAAR, P. J. 2022. Prehospital triage of patients with suspected non-ST-segment elevation acute coronary syndrome: Rationale and design of the TRIAGE-ACS study. *Contemporary clinical trials,* 119**,** 106854.

ISHAK, M., ALI, D., FOKKERT, M. J., SLINGERLAND, R. J., DIKKESCHEI, B., TOLSMA, R. T., LICHTVELD, R. A., BRUINS, W., BOOMARS, R., BRUHEIM, K., VAN EENENNAAM, F., TIMMERS, L., VOSKUIL, M., DOEVENDANS, P. A., MOSTERD, A., HOES, A. W., TEN BERG, J. M. & VAN 'T HOF, A. W. J. 2015. Fast assessment and management of chest pain without ST-elevation in the pre-hospital gateway: Rationale and design. *European Heart Journal: Acute Cardiovascular Care,* 4**,** 129-136.

MARTIN-RODRIGUEZ, F., SANZ-GARCIA, A., CASTRO-PORTILLO, E., DELGADO-BENITO, J. F., DEL POZO VEGAS, C., ORTEGA RABBIONE, G., MARTIN-HERRERO, F., MARTIN-CONTY, J. L. & LOPEZ-IZQUIERDO, R. 2021. Prehospital troponin as a predictor of early clinical deterioration. *European Journal of Clinical Investigation,* 51**,** e13591.

MULLEN, M., CERRI, G., MURRAY, R., TALBOT, A., SANSEVERINO, A., MCCAHILL, P., MANGOLDS, V., VOLTURO, J., DARLING, C. & RESTUCCIA, M. 2014. Use of point-of-care lactate in the prehospital aeromedical environment. *Prehospital & Disaster Medicine,* 29**,** 200-3.

TOLSMA, R. T., FOKKERT, M. J., VAN DONGEN, D. N., BADINGS, E. A., VAN DER SLUIS, A., SLINGERLAND, R. J., VAN, T. R. E., OTTERVANGER, J. P. & VANT HOF, A. W. J. 2022. Referral decisions based on a pre-hospital HEART score in suspected non-ST-elevation acute coronary syndrome: Final results of the FamouS Triage study. *European Heart Journal: Acute Cardiovascular Care,* 11(2)**,** 160-169.

VAN DONGEN, D. N., BADINGS, E. A., FOKKERT, M. J., TOLSMA, R. T., VAN DER SLUIS, A., SLINGERLAND, R. J., VAN'T HOF, A. W. J. & OTTERVANGER, J. P. 2021. Pre-hospital versus hospital acquired HEART score for risk classification of suspected non ST-elevation acute coronary syndrome. *European Journal of Cardiovascular Nursing,* 20(1)**,** 40-47.

VAN DONGEN, D. N., FOKKERT, M. J., TOLSMA, R. T., BADINGS, E. A., VAN DER SLUIS, A., SLINGERLAND, R. J., VAN 'T HOF, A. W. J. & OTTERVANGER, J. P. 2018. Value of Prehospital Troponin Assessment in Suspected Non-ST-Elevation Acute Coronary Syndrome. *American Journal of Cardiology,* 122**,** 1610-1616.

VAN DONGEN, D. N., FOKKERT, M. J., TOLSMA, R. T., VAN DER SLUIS, A., SLINGERLAND, R. J., BADINGS, E. A., VAN 'T HOF, A. W. J. & OTTERVANGER, J. P. 2020a. Accuracy of pre-hospital HEART score risk classification using point of care versus high sensitive troponin in suspected NSTE-ACS. *American Journal of Emergency Medicine,* 38**,** 1616-1620.

VAN DONGEN, D. N., TOLSMA, R. T., FOKKERT, M. J., BADINGS, E. A., VAN DER SLUIS, A., SLINGERLAND, R. J., VAN 'T HOF, A. W., VAN 'T RIET, E. & OTTERVANGER, J. P. 2020b. Referral decisions based on a prehospital HEART score in suspected non-ST-elevation acute coronary syndrome: design of the FamouS Triage 3 study. *Future Cardiology,* 16**,** 217-226.

VAN DONGEN, D. N., TOLSMA, R. T., FOKKERT, M. J., BADINGS, E. A., VAN DER SLUIS, A., SLINGERLAND, R. J., VAN, T. H. A. W. J. & OTTERVANGER, J. P. 2020c. Pre-hospital risk assessment in suspected non-ST-elevation acute coronary syndrome: A prospective observational study. *European Heart Journal: Acute Cardiovascular Care,* 9**,** 5-7.

Observational studies (Single arm cohort studies and observations of use of point of care tests in the EMS)

COLLOPY, K. T., WESTMORELAND, A. & POWERS, W. F. 2022. Patient Care Alterations After Point-of-Care Laboratory Testing During Critical Care Transport. *Air Medical Journal,* 41**,** 370-375.

CORRAL TORRES, E., HERNANDEZ-TEJEDOR, A., SUAREZ BUSTAMANTE, R., DE ELIAS HERNANDEZ, R., CASADO FLOREZ, I. & SAN JUAN LINARES, A. 2020. Prognostic value of venous blood analysis at the start of CPR in non-traumatic out-of-hospital cardiac arrest: association with ROSC and the neurological outcome. *Critical care (London, England),* 24**,** 60.

DI SERIO, F., PETRONELLI, M. A. & SAMMARTINO, E. 2010. Laboratory testing during critical care transport: point-of-care testing in air ambulances. *Clinical Chemistry & Laboratory Medicine,* 48**,** 955-61.

EASTMAN, J., ALLEN, D., MUMMA, K., ALMOND, A. & THEILING, J. 2021. Point-of-Care Laboratory Data Collection During Critical Care Transport. *Air Medical Journal,* 40**,** 81-83.

FUZERY, A. K. & KOST, G. J. 2020. Point-of-Care Testing Practices, Failure Modes, and Risk-Mitigation Strategies in Emergency Medical Services Programs in the Canadian Province of Alberta. *Archives of Pathology & Laboratory Medicine,* 144**,** 1352-1371.

GALVAGNO, S. M., JR., SIKORSKI, R. A., FLOCCARE, D. J., ROCK, P., MAZZEFFI, M. A., DUBOSE, J. J., SCALEA, T. M., MILLER, C., RICHARDS, J. E., O'CONNOR, J. V., MACKENZIE, C. F. & HU, P. 2020. Prehospital Point of Care Testing for the Early Detection of Shock and Prediction of Lifesaving Interventions. *Shock,* 54**,** 710-716.

GRUEBL, T., PLOEGER, B., WRANZE-BIELEFELD, E., MUELLER, M., SCHMIDBAUER, W., KILL, C. & BETZ, S. 2021. Point-of-care testing in out-of-hospital cardiac arrest: a retrospective analysis of relevance and consequences. *Scandinavian Journal of Trauma, Resuscitation & Emergency Medicine,* 29**,** 128.

HEANEY, K., WHITING, K., PETLEY, L., FRY, I. & NEWTON, A. 2020. Point-of-care testing by paramedics using a portable laboratory: an evaluation. *Journal of Paramedic Practice,* 12**,** 100-108.

HILL, J., GOTHARD, D. M. & MCLEAN, M. M. 2020. Prehospital Blood Glucose Testing as a Predictor of Impending Hypotension in Adult Trauma Patients. *Air Medical Journal,* 39**,** 20-23.

HOLSTEIN, A., PLASCHKE, A., VOGEL, M. Y. & EGBERTS, E. H. 2003. Prehospital management of diabetic emergencies--a population-based intervention study. *Acta anaesthesiologica Scandinavica,* 47**,** 610-5.

ISHAK, M., ALI, D., FOKKERT, M. J., SLINGERLAND, R. J., TOLSMA, R. T., BADINGS, E., VAN DER SLUIS, A., VAN EENENNAAM, F., MOSTERD, A., TEN BERG, J. M. & VAN 'T HOF, A. W. J. 2018. Fast assessment and management of chest pain patients without ST-elevation in the pre-hospital gateway (FamouS Triage): ruling out a myocardial infarction at home with the modified HEART score. *European Heart Journal: Acute Cardiovascular Care,* 7**,** 102-110.

JOUFFROY, R., LEGUILLIER, T., GILBERT, B., TOURTIER, J. P., BLOCH-LAINE, E., ECOLLAN, P., BOUNES, V., BOULARAN, J., GUEYE-NGALGOU, P., NIVET-ANTOINE, V., BEAUDEUX, J. L. & VIVIEN, B. 2021. Prehospital lactate clearance is associated with reduced mortality in patients with septic shock. *American Journal of Emergency Medicine,* 46**,** 367-373.

JOUSI, M., REITALA, J., LUND, V., KATILA, A. & LEPPANIEMI, A. 2010. The role of pre-hospital blood gas analysis in trauma resuscitation. *World journal of emergency surgery : WJES,* 5**,** 10.

LIGHTOWLER, B., HODGE, A., PILBERY, R., BELL, F., BEST, P., HIRD, K., WALKER, A. & SNAITH, B. 2023. Venous blood point-of-care testing (POCT) for paramedics in urgent and emergency care: protocol for a single-site feasibility study (POCTPara). *British paramedic journal,* 8**,** 34-41.

MCPHERSON, M. 2019. Point-of-care blood tests in decision-making for people over 65 with acute frailty. *Journal of Paramedic Practice,* 11**,** 106-114.

NADIM, G., LAURSEN, C. B., PIETERSEN, P. I., WITTROCK, D., SØRENSEN, M. K., NIELSEN, L. B., RASMUSSEN, C. H., CHRISTENSEN, H. M., HELMERIK, S., JØRGENSEN, G., TITLESTAD, I. L., LASSEN, A. T. & MIKKELSEN, S. 2021. Prehospital emergency medical technicians can perform ultrasonography and blood analysis in prehospital evaluation of patients with chronic obstructive pulmonary disease: a feasibility study. *BMC Health Serv Res,* 21**,** 290.

NOVAK, A., CHERRY, J., ALI, N., SMITH, I., BOWEN, J., RAY, J., BLACK, J. J. M., CORNETT, R., TAYLOR, S., HAYWARD, G. & LASSERSON, D. 2022. Point-of-care blood testing with secondary care decision support for frail patients. *Journal of Paramedic Practice,* 14**,** 54-62.

RICHARDS, A., MUDDASSIR, M., SAMPSON, F., MACLACHLAN, L., MILLER, E., FITCHETT, J., BELL, F., IVAN, M., LILLIE, P., SAMSON, A. & EASOM, N. 2022. Evaluation of pre-hospital COVID-19 rapid antigen tests by paramedics and their use in a direct admission pathway. *Journal of Infection,* 85**,** e53-e55.

SÖDERQVIST, M., VIRTA, J. & KÄMÄRÄINEN, A. 2018. Substance Abuse Among Emergency Medical Service Patients: A Pilot Study on the Clinical Impact of an On-site Oral Fluid Screening Test. *Point of Care,* 17**,** 47-49.

SORENSEN, J. T., TERKELSEN, C. J., STEENGAARD, C., LASSEN, J. F., TRAUTNER, S., CHRISTENSEN, E. F., NIELSEN, T. T., BOTKER, H. E., ANDERSEN, H. R. & THYGESEN, K. 2011. Prehospital troponin T testing in the diagnosis and triage of patients with suspected acute myocardial infarction. *American Journal of Cardiology,* 107**,** 1436-40.

STOPYRA, J. P., SNAVELY, A. C., SMITH, L. M., HARRIS, R. D., NELSON, R. D., WINSLOW, J. E., ALSON, R. L., POMPER, G. J., RILEY, R. F., ASHBURN, N. P., HENDLEY, N. W., GADDY, J., WOODRUM, T., FORNAGE, L., CONNER, D., ALVAREZ, M., PFLUM, A., KOEHLER, L. E., MILLER, C. D. & MAHLER, S. A. 2020. Prehospital use of a modified HEART Pathway and point-of-care troponin to predict cardiovascular events. *PLoS ONE [Electronic Resource],* 15**,** e0239460.

STROTE, J., CLOYD, D., REA, T. & EISENBERG, M. 2005. The influence of emergency medical technician glucometry on paramedic involvement. *Prehospital Emergency Care,* 9**,** 318-21.

TRAN, N. K. & KOST, G. J. 2006. Worldwide point-of-care testing: compendiums of POCT for mobile, emergency, critical, and primary care and of infectious diseases tests. *Point of Care,* 5**,** 84-92.

WILKINSON-STOKES, M., RYAN, E., WILLIAMS, M., SPENCER, M., MARIA, S. M. S. & COLBECK, M. 2021. A comparison of australasian jurisdictional ambulance services' paramedic clinical practice guidelines series: Adult sepsis. *Australasian Journal of Paramedicine,* 18.

Qualitative research

CHRISTENSEN, H. M., PIETERSEN, P. I., LAURSEN, C. B., WITTROCK, D., NADIM, G., JORGENSEN, G., NIELSEN, L. B., SORENSEN, M. K., TITLESTAD, I. L., LASSEN, A. T. & MIKKELSEN, S. 2022. Patients' perspectives on point-of-care diagnostics and treatment by emergency medical technicians in acute COPD exacerbations: A qualitative study. *Scandinavian Journal of Trauma, Resuscitation & Emergency Medicine,* 30**,** 11.

GREEN, K., MICOCCI, M., HICKS, T., WINTER, A., MARTIN, J. E., SHINKINS, B., SHAW, L., PRICE, C., DAVIES, K. & ALLEN, J. A. 2022. Perceived feasibility, facilitators and barriers to incorporating point-of-care testing for SARS-CoV-2 into emergency medical services by ambulance service staff: a survey-based approach. *BMJ Open,* 12**,** e064038.

LIGHTOWLER, B., HODGE, A., PILBERY, R., BELL, F., BEST, P., HIRD, K., WALKER, A. & SNAITH, B. 2023. Venous blood point-of-care testing (POCT) for paramedics in urgent and emergency care: protocol for a single-site feasibility study (POCTPara). *British paramedic journal,* 8**,** 34-41.

SAMPSON, F. C., BELL, F., COSTER, J. E., MILLER, E. & EASOM, N. 2022. Stakeholder perspectives of piloting pre-hospital COVID-19 lateral flow testing and direct admissions pathway: exploring why well-received ideas have low uptake. *British Paramedic Journal,* 7**,** 15-25.

Surveys

BEYNON, C., ERK, A. G., POTZY, A., MOHR, S. & POPP, E. 2015. Point of care coagulometry in prehospital emergency care: an observational study. *Scandinavian Journal of Trauma, Resuscitation & Emergency Medicine,* 23**,** 58.

BLANCHARD, I. E., KOZICKY, R., DALGARNO, D., SIMMS, J., GOULDER, S., WILLIAMSON, T. S., BIESBROEK, S., PAGE, L., LEAMAN, K., SNOZYK, S., REDMAN, L., SPACKMAN, K., DOIG, C. J., LANG, E. S. & LAZARENKO, G. 2019. Community paramedic point of care testing: validity and usability of two commercially available devices. *BMC Emergency Medicine,* 19**,** 30.

CHRISTENSEN, H. M., PIETERSEN, P. I., LAURSEN, C. B., WITTROCK, D., NADIM, G., JORGENSEN, G., NIELSEN, L. B., SORENSEN, M. K., TITLESTAD, I. L., LASSEN, A. T. & MIKKELSEN, S. 2022. Patients' perspectives on point-of-care diagnostics and treatment by emergency medical technicians in acute COPD exacerbations: A qualitative study. *Scandinavian Journal of*

HARJOLA, P., MIRO, O., MARTIN-SANCHEZ, F. J., ESCALADA, X., FREUND, Y., PENALOZA, A., CHRIST, M., CONE, D. C., LARIBI, S., KUISMA, M., TARVASMAKI, T., HARJOLA, V. P. & GROUP, E.-A. S. 2020. Pre-hospital management protocols and perceived difficulty in diagnosing acute heart failure. *ESC heart failure,* 7**,** 289-296.

HEANEY, K., WHITING, K., PETLEY, L., FRY, I. & NEWTON, A. 2020. Point-of-care testing by paramedics using a portable laboratory: an evaluation. *Journal of Paramedic Practice,* 12**,** 100-108.

HOLSTEIN, A., PLASCHKE, A., SCHLIEKER, H. & EGBERTS, E. H. 2002. Structural and process quality in the management of diabetic emergencies in Germany. *International Journal for Quality in Health Care,* 14**,** 33-38.

HOLSTEIN, D. J. F., HOLSTEIN, J. D., FISCHER, D., MENDE, M., FRIER, B. M. & HOLSTEIN, A. 2022. Out-of-Hospital Management of Diabetic Emergencies in Germany: Structural and Process Quality. *Experimental and Clinical Endocrinology and Diabetes,* 130**,** 454-461.

LEECH, C. & CLARKE, E. 2022. Pre-hospital blood products and calcium replacement protocols in UK critical care services: A survey of current practice. *Resuscitation Plus,* 11**,** 100282.

YOUNGER, P. & MCCLELLAND, G. 2014. Evaluation of pre-hospital point-of-care testing for lactate in sepsis and trauma patients. *Journal of Paramedic Practice,* 6**,** 526-531.

Diagnostic test accuracy

ALGHAMDI, A., COOK, E., CARLTON, E., SIRIWARDENA, A., HANN, M., THOMPSON, A., FOULKES, A., PHILLIPS, J., COOPER, J., BELL, S., KIRBY, K., ROSSER, A. & BODY, R. 2019. PRe-hospital Evaluation of Sensitive TrOponin (PRESTO) Study: multicentre prospective diagnostic accuracy study protocol. *BMJ Open,* 9**,** e032834.

ALGHAMDI, A., HANN, M., CARLTON, E., COOPER, J. G., COOK, E., FOULKES, A., SIRIWARDENA, A. N., PHILLIPS, J., THOMPSON, A., BELL, S., KIRBY, K., ROSSER, A. & BODY, R. 2023. Diagnostic Accuracy of Clinical Pathways for Suspected Acute Myocardial Infarction in the Out-of-Hospital Environment. *Annals of emergency medicine*.

BAEZ, A. A. & COCHON, L. 2016a. Acute Care Diagnostics Collaboration: Assessment of a Bayesian clinical decision model integrating the Prehospital Sepsis Score and point-of-care lactate. *American Journal of Emergency Medicine,* 34**,** 193-6.

BAEZ, A. A. & COCHON, L. 2016b. Acute care diagnostics collaboration: Bayesian diagnostic quality assessment of quick sepsis-related organ failure assessment score (qSOFA) integrated with out-of-hospital point-of-care lactate. *Annals of Emergency Medicine,* 68(4 Supplement 1)**,** S46.

BAEZ, A. A., LOPEZ, O., MARTINEZ, M. D. P., LIBELL, N., COCHON, L. & NICOLAS, J. M. 2021. Clinical validation demonstrates concordance of qSOFA and POC lactate Bayesian model: Results from the ACDC Phase-2 program. *American Journal of Emergency Medicine,* 45**,** 490-494.

BEYNON, C., ERK, A. G., POTZY, A., MOHR, S. & POPP, E. 2015. Point of care coagulometry in prehospital emergency care: an observational study. *Scandinavian Journal of Trauma, Resuscitation & Emergency Medicine,* 23**,** 58.

BODNAR, D., PARKER, L., MEISTER, M., RYAN, G., RASHFORD, S., WULLSCHLEGER, M., LAM, A. K. & BOSLEY, E. 2022. Correlation of prehospital point-of-care international normalized ratio to laboratory-based international normalized ratio in acute traumatic coagulopathy. *Journal of Trauma and Acute Care Surgery,* 92(6)**,** E127-E131.

BOLAND, L. L., HOKANSON, J. S., FERNSTROM, K. M., KINZY, T. G., LICK, C. J., SATTERLEE, P. A. & LACROIX, B. K. 2016. Prehospital Lactate Measurement by Emergency Medical Services in Patients Meeting Sepsis Criteria. *The western journal of emergency medicine,* 17**,** 648-55.

BROWN, J. B., LERNER, E. B., SPERRY, J. L., BILLIAR, T. R., PEITZMAN, A. B. & GUYETTE, F. X. 2016. Prehospital lactate improves accuracy of prehospital criteria for designating trauma activation level. *The journal of trauma and acute care surgery,* 81**,** 445-52.

CASTRO-PORTILLO, E., LOPEZ-IZQUIERDO, R., SANZ-GARCIA, A., ORTEGA, G. J., DELGADO-BENITO, J. F., CASTRO VILLAMOR, M. A., SANCHEZ-SOBERON, I., DEL POZO VEGAS, C. & MARTIN-RODRIGUEZ, F. 2022. Role of prehospital point-of-care N-terminal pro-brain natriuretic peptide in acute life-threatening cardiovascular disease. *International Journal of Cardiology,* 364**,** 126-132.

CHARLTON, K. & MOORE, H. 2021. PaRamEDIc assessment of laCTate in OHCA and survival to hospital (PREDICT protocol). *Journal of Paramedic Practice,* 13**,** 100-104.

COOPER, J. G., FERGUSON, J., DONALDSON, L. A., BLACK, K. M. M., LIVOCK, K. J., HORRILL, J. L., DAVIDSON, E. M., SCOTT, N. W., LEE, A. J., FUJISAWA, T., LEE, K. K., ANAND, A., SHAH, A. S. V. & MILLS, N. L. 2021. The Ambulance Cardiac Chest Pain Evaluation in Scotland Study (ACCESS): A Prospective Cohort Study. *Annals of Emergency Medicine,* 77**,** 575-588.

DI SERIO, F., LOVERO, R., LEONE, M., DE SARIO, R., RUGGIERI, V., VARRASO, L. & PANSINI, N. 2006. Integration between the tele-cardiology unit and the central laboratory: methodological and clinical evaluation of point-of-care testing cardiac marker in the ambulance. *Clinical Chemistry & Laboratory Medicine,* 44**,** 768-73.

ECOLLAN, P., COLLET, J. P., BOON, G., TANGUY, M. L., FIEVET, M. L., HAAS, R., BERTHO, N., SIAMI, S., HUBERT, J. C., CORIAT, P. & MONTALESCOT, G. 2007. Pre-hospital detection of acute myocardial infarction with ultra-rapid human fatty acid-binding protein (H-FABP) immunoassay. *International Journal of Cardiology,* 119(3)**,** 349-354.

FUKUMA, H., NAKADA, T.-A., SHIMADA, T., SHIMAZUI, T., AIZIMU, T., NAKAO, S., WATANABE, H., MIZUSHIMA, Y. & MATSUOKA, T. 2019. Prehospital lactate improves prediction of the need for immediate interventions for hemorrhage after trauma. *Scientific reports,* 9**,** 13755.

GALVAGNO, S. M., JR., SIKORSKI, R. A., FLOCCARE, D. J., ROCK, P., MAZZEFFI, M. A., DUBOSE, J. J., SCALEA, T. M., MILLER, C., RICHARDS, J. E., O'CONNOR, J. V., MACKENZIE, C. F. & HU, P. 2020. Prehospital Point of Care Testing for the Early Detection of Shock and Prediction of Lifesaving Interventions. *Shock,* 54**,** 710-716.

GRIGGS, J. E., LYON, R. M., SHERRIFF, M., BARRETT, J. W., WAREHAM, G. & TER AVEST, E. 2022. Predictive clinical utility of pre-hospital point of care lactate for transfusion of blood product in patients with suspected traumatic haemorrhage: derivation of a decision-support tool. *Scandinavian journal of trauma, resuscitation and emergency medicine,* 30**,** 72.

GUERRA, W. F., MAYFIELD, T. R., MEYERS, M. S., CLOUATRE, A. E. & RICCIO, J. C. 2013. Early detection and treatment of patients with severe sepsis by prehospital personnel. *Journal of Emergency Medicine,* 44**,** 1116-25.

GUYETTE, F., SUFFOLETTO, B., CASTILLO, J.-L., QUINTERO, J., CALLAWAY, C. & PUYANA, J.-C. 2011. Prehospital serum lactate as a predictor of outcomes in trauma patients: a retrospective observational study. *The Journal of trauma,* 70**,** 782-6.

GUYETTE, F. X., MEIER, E. N., NEWGARD, C., MCKNIGHT, B., DAYA, M., BULGER, E. M., POWELL, J. L., BRASEL, K. J., KERBY, J. D., EGAN, D., SISE, M., COIMBRA, R., FABIAN, T. C., HOYT, D. B. & INVESTIGATORS, R. O. C. 2015. A comparison of prehospital lactate and systolic blood pressure for predicting the need for resuscitative care in trauma transported by ground. *The Journal of Trauma and Acute Care Surgery,* 78**,** 600-6.

HILL, J., GOTHARD, D. M. & MCLEAN, M. M. 2020. Prehospital Blood Glucose Testing as a Predictor of Impending Hypotension in Adult Trauma Patients. *Air Medical Journal,* 39**,** 20-23.

ISHAK, M., ALI, D., FOKKERT, M. J., SLINGERLAND, R. J., TOLSMA, R. T., BADINGS, E., VAN DER SLUIS, A., VAN EENENNAAM, F., MOSTERD, A., TEN BERG, J. M. & VAN 'T HOF, A. W. J. 2018. Fast assessment and management of chest pain patients without ST-elevation in the pre-hospital gateway (FamouS Triage): ruling out a myocardial infarction at home with the modified HEART score. *European Heart Journal: Acute Cardiovascular Care,* 7**,** 102-110.

JACOBSEN, L., GRENNE, B., OLSEN, R. B. & JORTVEIT, J. 2022. Feasibility of prehospital identification of non-ST-elevation myocardial infarction by ECG, troponin and echocardiography. *Emergency Medicine Journal,* 21**,** 21.

JANSEN, T. C., VAN BOMMEL, J., MULDER, P. G., ROMMES, J. H., SCHIEVELD, S. J. M. & BAKKER, J. 2008. The prognostic value of blood lactate levels relative to that of vital signs in the pre-hospital setting: a pilot study. *Critical care (London, England),* 12**,** R160.

JOUFFROY, R., LEGUILLIER, T., GILBERT, B., TOURTIER, J. P., BLOCH-LAINE, E., ECOLLAN, P., BOUNES, V., BOULARAN, J., GUEYE-NGALGOU, P., NIVET-ANTOINE, V., BEAUDEUX, J.-L. & VIVIEN, B. 2020a. Pre-Hospital Lactatemia Predicts 30-Day Mortality in Patients with Septic Shock-Preliminary Results from the LAPHSUS Study. *Journal of clinical medicine,* 9.

JOUFFROY, R., LEGUILLIER, T., GILBERT, B., TOURTIER, J. P., BLOCH-LAINE, E., ECOLLAN, P., BOUNES, V., BOULARAN, J., GUEYE-NGALGOU, P., NIVET-ANTOINE, V., BEAUDEUX, J. L. & VIVIEN, B. 2021. Prehospital lactate clearance is associated with reduced mortality in patients with septic shock. *American Journal of Emergency Medicine,* 46**,** 367-373.

JOUFFROY, R., TOURTIER, J. P., DEBATY, G., BOUNES, V., GUEYE-NGALGOU, P. & VIVIEN, B. 2020b. Contribution of the Pre-Hospital Blood Lactate Level in the Pre-Hospital Orientation of Septic Shock: The LAPHSUS Study. *Turkish journal of anaesthesiology and reanimation,* 48**,** 58-61.

KLEMEN, P., GOLUB, M. & GRMEC, S. 2009. Combination of quantitative capnometry, N-terminal pro-brain natriuretic peptide, and clinical assessment in differentiating acute heart failure from pulmonary disease as cause of acute dyspnea in pre-hospital emergency setting: study of diagnostic accuracy. *Croatian medical journal,* 50**,** 133-42.

MAGNUSSON, C., HERLITZ, J., HOGLIND, R., WENNBERG, P., EDELVIK TRANBERG, A., AXELSSON, C. & ZELANO, J. 2021. Prehospital lactate levels in blood as a seizure biomarker: A multi-center observational study. *Epilepsia,* 62(2)**,** 408-415.

MARTIN-RODRIGUEZ, F., LOPEZ-IZQUIERDO, R., CASTRO VILLAMOR, M. A., DEL POZO VEGAS, C., DELGADO BENITO, M. D. P., MARTINEZ CABALLERO, C. M., PRIEGO MARTINEZ, V., MARTIN CONTY, J. L., MAYO-ISCAR, A., SANCHEZ-SOBERON, I. & BAEZ, G. P. 2020a. The Prognostic Value of Prehospital Blood Lactate Levels to Predict Early Mortality in Acute Cardiovascular Disease. *Shock,* 53(2)**,** 164-170.

MARTIN-RODRIGUEZ, F., LOPEZ-IZQUIERDO, R., CASTRO VILLAMOR, M. A., MANGAS, I. M., DEL BRIO IBANEZ, P., DELGADO BENITO, J. F., MARTIN CONTY, J. L., MANZANARES, J. A., MAYO-ISCAR, A. & DEL POZO VEGAS, C. 2019. Prognostic value of lactate in prehospital care as a predictor of early mortality. *The American journal of emergency medicine,* 37**,** 1627-1632.

MARTIN-RODRIGUEZ, F., LOPEZ-IZQUIERDO, R., DELGADO BENITO, J. F., SANZ-GARCIA, A., DEL POZO VEGAS, C., CASTRO VILLAMOR, M. A., MARTIN-CONTY, J. L. & ORTEGA, G. J. 2020b. Prehospital Point-Of-Care Lactate Increases the Prognostic Accuracy of National Early Warning Score 2 for Early Risk Stratification of Mortality: Results of a Multicenter, Observational Study. *Journal of Clinical Medicine,* 9**,** 18.

MARTIN-RODRIGUEZ, F., LOPEZ-IZQUIERDO, R., MEDINA-LOZANO, E., ORTEGA RABBIONE, G., DEL POZO VEGAS, C., CARBAJOSA RODRIGUEZ, V., CASTRO VILLAMOR, M. A., SANCHEZ-SOBERON, I. & SANZ-GARCIA, A. 2020c. Accuracy of prehospital point-of-care lactate in early in-hospital mortality. *European Journal of Clinical Investigation,* 50**,** e13341.

MARTIN-RODRIGUEZ, F., MELERO-GUIJARRO, L., ORTEGA, G. J., SANZ-GARCIA, A., DE LA TORRE DE DIOS, T., MANZANARES, J. A., MARTIN-CONTY, J. L., CASTRO VILLAMOR, M. A., DELGADO BENITO, J. F. & LOPEZ-IZQUIERDO, R. 2022. Combination of Prehospital NT-proBNP with qSOFA and NEWS to Predict Sepsis and Sepsis-Related Mortality. *Disease markers,* 2022**,** 5351137.

MARTIN-RODRIGUEZ, F., SANZ-GARCIA, A., MARTINEZ FERNANDEZ, F. T., OTERO DE LA TORRE, S., DELGADO BENITO, J. F., DEL POZO VEGAS, C., GARCIA, R. P., ASTORGA, E. A. I., COALLA, A. S. & LOPEZ-IZQUIERDO, R. 2023a. Association between prehospital lactate categories with short- and long-term mortality. A prospective, observational multicenter study. *QJM : monthly journal of the Association of Physicians*.

MARTIN-RODRIGUEZ, F., VAQUERIZO-VILLAR, F., LOPEZ-IZQUIERDO, R., CASTRO-VILLAMOR, M. A., SANZ-GARCIA, A., DEL POZO-VEGAS, C. & HORNERO, R. 2023b. Derivation and validation of a blood biomarker score for 2-day mortality prediction from prehospital care: a multicenter, cohort, EMS-based study. *Internal and emergency medicine*.

MELERO-GUIJARRO, L., SANZ-GARCIA, A., MARTIN-RODRIGUEZ, F., LIPARI, V., MAZAS PEREZ OLEAGA, C., CARVAJAL ALTAMIRANDA, S., MARTINEZ LOPEZ, N. M., DOMINGUEZ AZPIROZ, I., CASTRO VILLAMOR, M. A., SANCHEZ SOBERON, I. & LOPEZ-IZQUIERDO, R. 2023. Prehospital qSOFA, mSOFA, and NEWS2 performance for sepsis prediction: A prospective, multi-center, cohort study. *Frontiers in medicine,* 10**,** 1149736.

NORA, M., DERI, D., VERES, D. S., KIS, Z., BARCSAY, E. & PALYI, B. 2022. Evaluating the field performance of multiple SARS-Cov-2 antigen rapid tests using nasopharyngeal swab samples. *PLoS ONE [Electronic Resource],* 17**,** e0262399.

OLANDER, A., MAGNUSSON, C., SUNDLER, A. J., BREMER, A., ANDERSSON, H., HERLITZ, J., AXELSSON, C. & ANDERSSON HAGIWARA, M. 2023. Prediction of the Risk of Sepsis by Using Analysis of Plasma Glucose and Serum Lactate in Ambulance Services: A Prospective Study. *Prehospital and disaster medicine,* 38**,** 160-167.

PAVLOVSKY, T., OBADIA, M., RAGOT, S., DOUAY, B., CASALINO, E. & GHAZALI, D. A. 2022. Predictors of Risk Stratification and Value of Point-of-Care of High-Sensitivity Cardiac Troponin-I in EMS Management of Non-ST-Segment Elevation Myocardial Infarction: A Retrospective Study. *Prehospital and disaster medicine,* 37**,** 365-372.

PHILLIPS, J., FRYER, T. L., BERNS, K. S., WOCKENFUS, A. M., SORENSON, L. A., SANTRACH, P. J. & ZIETLOW, S. P. 2021. Validation of a Point-of-Care Analyzer for Determining Anticoagulation Status During Air Transport. *Air Medical Journal,* 40**,** 322-324.

RASMUSSEN, M. B., STENGAARD, C., SORENSEN, J. T., RIDDERVOLD, I. S., HANSEN, T. M., GIEBNER, M., RASMUSSEN, C. H., BOTKER, H. E. & TERKELSEN, C. J. 2019. Predictive value of routine point-of-care cardiac troponin T measurement for prehospital diagnosis and risk-stratification in patients with suspected acute myocardial infarction. *European Heart Journal: Acute Cardiovascular Care,* 8**,** 299-308.

RICHARDS, A., MUDDASSIR, M., SAMPSON, F., MACLACHLAN, L., MILLER, E., FITCHETT, J., BELL, F., IVAN, M., LILLIE, P., SAMSON, A. & EASOM, N. 2022. Evaluation of pre-hospital COVID-19 rapid antigen tests by paramedics and their use in a direct admission pathway. *Journal of Infection,* 85**,** e53-e55.

SAGEL, D., VLAAR, P. J., VAN ROOSMALEN, R., WAARDENBURG, I., NIEUWLAND, W., LETTINGA, R., VAN BARNEVELD, R., JORNA, E., KIJLSTRA, R., VAN WELL, C., OOMEN, A., BARTELS, L., ANTHONIO, R., HAGENS, V., HOFMA, S., GU, Y., DRENTH, D., ADDINK, R., VAN ASSELT, T., VAN DER MEER, P., LIPSIC, E., JUAREZ OROZCO, L. & VAN DER HARST, P. 2021. Prehospital risk stratification in patients with chest pain. *Emergency Medicine Journal,* 38**,** 814-819.

SHAW, L., GRAZIADIO, S., LENDREM, C., DALE, N., FORD, G. A., ROFFE, C., SMITH, C. J., WHITE, P. M. & PRICE, C. I. 2021. Purines for Rapid Identification of Stroke Mimics (PRISM): study protocol for a diagnostic accuracy study. *Diagnostic and Prognostic Research,* 5**,** 11.

SORENSEN, J. T., TERKELSEN, C. J., STEENGAARD, C., LASSEN, J. F., TRAUTNER, S., CHRISTENSEN, E. F., NIELSEN, T. T., BOTKER, H. E., ANDERSEN, H. R. & THYGESEN, K. 2011. Prehospital troponin T testing in the diagnosis and triage of patients with suspected acute myocardial infarction. *American Journal of Cardiology,* 107**,** 1436-40.

ST JOHN, A. E., MCCOY, A. M., MOYES, A. G., GUYETTE, F. X., BULGER, E. M. & SAYRE, M. R. 2018. Prehospital Lactate Predicts Need for Resuscitative Care in Non-hypotensive Trauma Patients. *The western journal of emergency medicine,* 19**,** 224-231.

STENGAARD, C., SORENSEN, J. T., LADEFOGED, S. A., CHRISTENSEN, E. F., LASSEN, J. F., BOTKER, H. E., TERKELSEN, C. J. & THYGESEN, K. 2013. Quantitative point-of-care troponin T measurement for diagnosis and prognosis in patients with a suspected acute myocardial infarction. *American Journal of Cardiology,* 112**,** 1361-6.

STENGAARD, C., SORENSEN, J. T., LADEFOGED, S. A., LASSEN, J. F., RASMUSSEN, M. B., PEDERSEN, C. K., AYER, A., BOTKER, H. E., TERKELSEN, C. J. & THYGESEN, K. 2017. The potential of optimizing prehospital triage of patients with suspected acute myocardial infarction using high-sensitivity cardiac troponin T and copeptin. *Biomarkers,* 22**,** 351-360.

STOLL, D., ENGLUND, E., HILLBORG, H., VEDIN, S. & LARSSON, A. 2018. Capillary and venous lactate measurements with a handheld device compared to venous blood-gas analysis for emergency patients. *Scandinavian Journal of Trauma, Resuscitation & Emergency Medicine,* 26**,** 47.

STOPYRA, J. P., SNAVELY, A. C., SCHEIDLER, J. F., SMITH, L. M., NELSON, R. D., WINSLOW, J. E., POMPER, G. J., ASHBURN, N. P., HENDLEY, N. W., RILEY, R. F., KOEHLER, L. E., MILLER, C. D. & MAHLER, S. A. 2020. Point-of-Care Troponin Testing during Ambulance Transport to Detect Acute Myocardial Infarction. *Prehospital Emergency Care,* 24**,** 751-759.

SVENSSON, L., AXELSSON, C., NORDLANDER, R. & HERLITZ, J. 2003. Elevation of biochemical markers for myocardial damage prior to hospital admission in patients with acute chest pain or other symptoms raising suspicion of acute coronary syndrome. *Journal of Internal Medicine,* 253**,** 311-9.

SVENSSON, L., AXELSSON, C., NORDLANDER, R. & HERLITZ, J. 2004. Prognostic value of biochemical markers, 12-lead ECG and patient characteristics amongst patients calling for an ambulance due to a suspected acute coronary syndrome. *Journal of Internal Medicine,* 255**,** 469-77.

SWAN, K. L., AVARD, B. J. & KEENE, T. 2019. The relationship between elevated prehospital point-of-care lactate measurements, intensive care unit admission, and mortality: A retrospective review of adult patients. *Australian Critical Care,* 32**,** 100-105.

SWAN, K. L., KEENE, T. & AVARD, B. J. 2018. A 12-Month Clinical Audit Comparing Point-of-Care Lactate Measurements Tested by Paramedics with In-Hospital Serum Lactate Measurements. *Prehospital & Disaster Medicine,* 33**,** 36-42.

TEBOUL, A., GAFFINEL, A., MEUNE, C., GREFFET, A., SAUVAL, P. & CARLI, P. 2004. Management of acute dyspnoea: use and feasibility of brain natriuretic peptide (BNP) assay in the prehospital setting. *Resuscitation,* 61**,** 91-6.

TOBIAS, A. Z., GUYETTE, F. X., SEYMOUR, C. W., SUFFOLETTO, B. P., MARTIN-GILL, C., QUINTERO, J., KRISTAN, J., CALLAWAY, C. W. & YEALY, D. M. 2014. Pre-resuscitation lactate and hospital mortality in prehospital patients. *Prehospital emergency care,* 18**,** 321-7.

VAN BEEST, P. A., MULDER, P. J., OETOMO, S. B., VAN DEN BROEK, B., KUIPER, M. A. & SPRONK, P. E. 2009. Measurement of lactate in a prehospital setting is related to outcome. *European journal of emergency medicine : official journal of the European Society for Emergency Medicine,* 16**,** 318-22.

VAN DER WAARDEN, N. W. P. L., SCHOTTING, B., ROYAARDS, K. J., VLACHOJANNIS, G. & BACKUS, B. E. 2022. Reliability of the HEART-score in the prehospital setting using point-of-care troponin. *European Journal of Emergency Medicine,* 29**,** 450-451.

WALLGREN, U. M., SJOLIN, J., JARNBERT-PETTERSSON, H. & KURLAND, L. 2020. The predictive value of variables measurable in the ambulance and the development of the Predict Sepsis screening tools: a prospective cohort study. *Scandinavian Journal of Trauma, Resuscitation & Emergency Medicine,* 28**,** 59.

WALTHER, L. H., ZEGERS, F., NYBO, M., MOGENSEN, C. B., CHRISTENSEN, E. F., LASSEN, A. T. & MIKKELSEN, S. 2022. Accuracy of a point-of-care blood lactate measurement device in a prehospital setting. *Journal of Clinical Monitoring & Computing,* 27**,** 27.

YOUNGER, P. & MCCLELLAND, G. 2014. Evaluation of pre-hospital point-of-care testing for lactate in sepsis and trauma patients. *Journal of Paramedic Practice,* 6**,** 526-531.

Developmental research

BATES, A., DONOHUE, A., MCCULLOUGH, J. & WINEARLS, J. 2020. Viscoelastic haemostatic assays in aeromedical transport. *Emergency Medicine Australasia,* 32**,** 786-792.

BLANCHARD, I. E., KOZICKY, R., DALGARNO, D., SIMMS, J., GOULDER, S., WILLIAMSON, T. S., BIESBROEK, S., PAGE, L., LEAMAN, K., SNOZYK, S., REDMAN, L., SPACKMAN, K., DOIG, C. J., LANG, E. S. & LAZARENKO, G. 2019. Community paramedic point of care testing: validity and usability of two commercially available devices. *BMC Emergency Medicine,* 19**,** 30.

BOYE, M., BOISSIN, J., POYAT, C., PASQUIER, P. & MARTINAUD, C. 2022. Evaluation of the altitude impact on a point-of-care thromboelastography analyzer measurement: prerequisites for use in airborne medical evacuation courses. *European Journal of Trauma & Emergency Surgery,* 48**,** 489-495.

COLON-FRANCO, J. M., LO, S. F., TARIMA, S. S., GOURLAY, D., DRENDEL, A. L. & BROOK LERNER, E. 2017. Validation of a hand-held point of care device for lactate in adult and pediatric patients using traditional and locally-smoothed median and maximum absolute difference curves. *Clinica Chimica Acta,* 468**,** 145-149.

KARON, B. S., SCOTT, R., BURRITT, M. F. & SANTRACH, P. J. 2007. Comparison of lactate values between point-of-care and central laboratory analyzers. *American journal of clinical pathology,* 128**,** 168-71.

LEGUILLIER, T., JOUFFROY, R., BOISSON, M., BOUSSAROQUE, A., CHENEVIER-GOBEAUX, C., CHAABOUNI, T., VIVIEN, B., NIVET-ANTOINE, V. & BEAUDEUX, J.-L. 2018. Lactate POCT in mobile intensive care units for septic patients? A comparison of capillary blood method versus venous blood and plasma-based reference methods. *Clinical biochemistry,* 55**,** 9-14.

LIU, H.-L., TSENG, Y.-T., LAI, M.-C. & CHAU, L.-K. 2022. Ultrasensitive and Rapid Detection of N-Terminal Pro-B-Type Natriuretic Peptide (NT-proBNP) Using Fiber Optic Nanogold-Linked Immunosorbent Assay. *Biosensors,* 12.

MIKKELSEN, S., WOLSING-HANSEN, J., NYBO, M., MAEGAARD, C. U. & JEPSEN, S. 2015. Implementation of the ABL-90 blood gas analyzer in a ground-based mobile emergency care unit. *Scandinavian Journal of Trauma, Resuscitation & Emergency Medicine,* 23**,** 54.

SCHOBER, P., BOSSERS, S. M., KOOLWIJK, J., TERRA, M. & SCHWARTE, L. A. 2021. Prehospital coagulation measurement by a portable blood analyzer in a helicopter emergency medical service (HEMS). *American Journal of Emergency Medicine,* 46**,** 137-140.

SCHOBER, P., BOSSERS, S. M., KRAGE, R., DE LEEUW, M. A. & SCHWARTE, L. A. 2019. Portable Blood (Gas) Analyzer in a Helicopter Emergency Medical Service. *Air Medical Journal,* 38**,** 302-304.

SCOTT, H. F., DONOGHUE, A. J., GAIESKI, D. F., MARCHESE, R. F. & MISTRY, R. D. 2012. The utility of early lactate testing in undifferentiated pediatric systemic inflammatory response syndrome. *Academic Emergency Medicine,* 19**,** 1276-1280.

TER AVEST, E., GRIGGS, J., WIJESURIYA, J., RUSSELL, M. Q. & LYON, R. M. 2020. Determinants of prehospital lactate in trauma patients: a retrospective cohort study. *BMC Emergency Medicine,* 20**,** 18.

TOPPING, J., REARDON, M., COLEMAN, J., HUNTER, B., SHOJIMA-PERERA, H., THYER, L. & SIMPSON, P. 2019. A Comparison of Venous versus Capillary Blood Samples when Measuring Blood Glucose Using a Point-of-Care, Capillary-Based Glucometer. *Prehospital & Disaster Medicine,* 34**,** 506-509.

VAN DEN BROEK, J., BISCHOF, D., DERRON, N., ABEGG, S., GERBER, P. A., GUNTNER, A. T. & PRATSINIS, S. E. 2021. Screening Methanol Poisoning with a Portable Breath Detector. *Analytical Chemistry,* 93**,** 1170-1178.

VENTURINI, J. M., STAKE, C. E. & CICHON, M. E. 2013. Prehospital point-of-care testing for troponin: are the results reliable? *Prehospital Emergency Care,* 17**,** 88-91.

ZIPPERLE, J., ZIEGLER, B., SCHOCHL, H., VOELCKEL, W., SCHLIMP, C. J. & OBERLADSTATTER, D. 2022. Operability of a Resonance-Based Viscoelastic Haemostatic Analyzer in the High-Vibration Environment of Air Medical Transport. *Journal of Clinical Medicine,* 11**,** 23.
